# 5-Fluorouracil in Combination with Calcium Carbonate Nanoparticles Loaded with Antioxidant Thymoquinone against Colon Cancer: Synergistically Therapeutic Potential and Underlying Molecular Mechanism

**DOI:** 10.3390/antiox13091030

**Published:** 2024-08-25

**Authors:** Xi Deng, Zhongming Yang, Kim Wei Chan, Norsharina Ismail, Md Zuki Abu Bakar

**Affiliations:** 1 Natural Medicines and Products Research Laboratory, Institute of Bioscience, Universiti Putra Malaysia, Serdang 43400, Selangor, Malaysia; dengxi9528@126.com (X.D.); yzm719268164@gmail.com (Z.Y.); chankim@upm.edu.my (K.W.C.); norsharina@upm.edu.my (N.I.); 2 Department of Veterinary Preclinical Science, Faculty of Veterinary Medicine, Universiti Putra Malaysia, Serdang 43400, Selangor, Malaysia

**Keywords:** 5-fluorouracil, thymoquinone-loaded calcium carbonate nanoparticles, synthesis and characterization, colon cancer, synergistically therapeutic effects, molecular therapeutic mechanisms

## Abstract

Colon cancer is the third most common cancer worldwide, with high mortality. Adverse side effects and chemoresistance of the first-line chemotherapy 5-fluorouracil (5-FU) have promoted the widespread use of combination therapies. Thymoquinone (TQ) is a natural compound with potent antioxidant activity. Loading antioxidants into nano delivery systems has been a major advance in enhancing their bioavailability to improve clinical application. Hence, this study aimed to prepare the optimal TQ-loaded calcium carbonate nanoparticles (TQ-CaCO_3_ NPs) and investigate their therapeutic potential and underlying molecular mechanisms of TQ-CaCO_3_ NPs in combination with 5-FU against colon cancer. Firstly, we developed purely aragonite CaCO_3_ NPs with a facile mechanical ball-milling method. The pH-sensitive and biocompatible TQ-CaCO_3_ NPs with sustained release properties were prepared using the optimal synthesized method (a high-speed homogenizer). The in vitro study revealed that the combination of TQ-CaCO_3_ NPs (15 μM) and 5-FU (7.5 μM) inhibited CT26 cell proliferation and migration, induced cell apoptosis and cell cycle arrest in the G_0_/G_1_ phase, and suppressed the CT26 spheroid growth, exhibiting a synergistic effect. Finally, network pharmacology and molecular docking results indicated the potential targets and crucial signaling pathways of TQ-CaCO_3_ NPs in combination with 5-FU against colon cancer. Therefore, TQ-CaCO_3_ NPs combined with 5-FU could enhance the anti-colon cancer effects of 5-FU with broader therapeutic targets, warranting further application for colon cancer treatment.

## 1. Introduction

According to the latest global cancer statistics in the *GLOBOCAN* database, colon cancer remains one of the major causes of morbidity and mortality worldwide, which is largely attributed to a relatively higher intake of animal-source foods and an increasingly sedentary lifestyle [1]. Given a constant incidence rate, the expanding and aging population will lead to a significant rise in cases of colon cancer, imposing a substantial burden on society. 5-Fluorouracil (5-FU) is one of the commonly used chemotherapy drugs in the treatment of colon cancer. However, insufficient accumulation of 5-FU in tumors due to non-specificity and a low plasma half-life, as well as limited response rates (10–15%), results in high-dosage uses of 5-FU, thereby leading to severe toxic side effects such as nausea, diarrhea, fever, myocardial ischemia, and neutropenia [2,3]. Therefore, there is an urgent need to explore new chemotherapy synergistic drugs, reducing the dose of chemotherapy drugs or shortening the chemotherapy cycle while maintaining the effects of chemotherapy, to improve the life quality of patients and minimize toxic side effects. 

Thymoquinone (TQ), the primary active ingredient of the natural product *Nigella sativa* seeds, is a promising natural antioxidant. It acts by scavenging free radicals, thereby reducing oxidative stress. TQ enhances the activity of antioxidant enzymes such as superoxide dismutase, catalase, and glutathione peroxidase, which protect cells from oxidative damage [4]. Additionally, TQ inhibits lipid peroxidation, a process that damages cell membranes and leads to cell death [5]. It exhibits preventive or therapeutic effects on osteosarcoma, colon cancer, and gastric cancer through modulating reactive oxygen species production and cellular proliferation, autophagy, apoptosis, invasion, and metastasis [6,7,8]. Importantly, the combinatorial uses of the antioxidant TQ and chemotherapy drug 5-FU have shown promising anticancer properties in the treatment of triple-negative breast cancer and gastric cancer [9,10]. However, the underlying mechanisms of TQ in combination with 5-FU against cancer have not been comprehensively investigated. Moreover, the high hydrophobic property of TQ contributes to its poor solubility and low bioavailability, leading to limited therapeutic effects [11,12]. To improve the anticancer efficacy of TQ while reducing toxic side effects, various nanocarriers have been applied to deliver it to target cancerous cells. For example, PEGylated-TQ nanoparticles and TQ-loaded poly lactic-co-glycolic acid nanoparticles were synthesized for breast cancer and malignant melanoma treatment, respectively [13,14]. 

The ideal anti-cancer drug delivery system should have the ability of favorable sustained release and pronounced biocompatibility with simple and economical synthesis methods [15,16]. Thus, it can deliver the drugs to target sites to enhance the anti-cancer efficacy of drugs and reduce their side effects, with no or low toxicity to normal tissues and cells. Calcium carbonate has been approved for clinical use by the U.S. Food and Drug Administration (FDA). Calcium carbonate nanoparticles have been widely studied as nanocarriers of drugs against cancer due to their excellent biocompatibility, pH responsiveness, and biodegradability. As reported, calcium carbonate nanoparticles showed safety on the normal breast cell line MCF10A, fibroblasts NIH3T3, normal tissues in C57BL/6 mice, as well as hematological and biochemical levels in Kunming mice, and satisfactory blood compatibility [17,18,19,20]. Moreover, calcium carbonate nanoparticles exhibited remarkable pH-controlled drug release, with lower release at pH 7.4 in the blood and healthy tissue environment but more rapid release in the acidic tumor environment [21,22,23,24]. Meanwhile, calcium carbonate nanoparticles can decompose into Ca^2+^ and CO_2_ under acidic conditions, facilitating tumor-targeted delivery [25]. Cockle shells, abundant in coastal areas, are natural sources of calcium carbonate that is low-cost and easily available. Moreover, cockle shells consist of good-quality calcium carbonate in the aragonite polymorph form, of which aragonite is a stable phase of calcium carbonate [26,27]. Furthermore, compared with down-top methods such as precipitation, emulsion, and polymerization, mechanical ball milling (top-down approach) can synthesize large-scale cockle shell-derived calcium carbonate nanoparticles by a simple operation while maintaining their unique features [28,29].

Herein, this study used a mechanical ball-milling approach to develop cockle shell-derived aragonite calcium carbonate nanoparticles (CaCO_3_ NPs) and synthesized TQ-loaded CaCO_3_ NPs (TQ-CaCO_3_ NPs) using the optimal formulation. Characterization of nanoparticles was performed to examine the potential of CaCO_3_ NPs for antioxidant TQ delivery. The combinatorial effects of TQ-CaCO_3_ NPs and 5-FU were then assessed in colon cancer in vitro models. Furthermore, the underlying therapeutic mechanisms of the combination were investigated by using network pharmacology and molecular docking. This study may present new insights into the combinatorial therapy for colon cancer treatment, with potential implications for future applications.

## 2. Materials and Methods

### 2.1. Materials

Cockle shells were sourced from the local market in Serdang, Malaysia. We also used dodecyl dimethyl betaine (BS-12, Jindun Industrial, Shanghai, China), thymoquinone (TQ, Molekula, Darlington, UK), and deionized water with a resistance of 18.2 MΩ/cm from an ELGA LabWater PURELAB flex3 water purification system (Type I) (ELGA, High Wycombe, UK). Brine shrimp (Artemia salina) was purchased from local pet shop in Kuala Lumpur, Malaysia; NIH3T3 embryonic fibroblast cell line and CT26 colon cancer cell line were sourced from the American Type Culture Collection (ATCC, Manassas, VA, USA); we also used RPMI-1640 with L-glutamine (Cytiva, Freiburg, Germany), fetal bovine serum (FBS, Cytiva, Freiburg, Germany), penicillin–streptomycin (Elabscience, Wuhan, China), trypsin-EDTA (1×) (Cytiva, Freiburg, Germany), 3-(4,5-dimethylthiazol-2-yl)-2,5-diphenyltetrazolium bromide) (MTT, Solarbio, Beijing, China), dimethylsulfoxide (DMSO, ChemAR, Kielce, Poland), 5-fluorouracil (5-FU, Molekula, Darlington, UK), crystal violet (Solarbio, Beijing, China), acridine orange (AO, Sigma-Aldrich, St. Louis, MO, USA), ribonuclease A (RNase A) and propidium iodide (PI) (Nacalai Tesque, Kyoto, Japan), and Annexin V-FITC apoptosis kit (BD Bioscience, Fremont, CA, USA). All other reagents were of analytical grade.

### 2.2. Preparation of CaCO_3_ NPs from Cockle Shells

The initial step was the preparation of micro-sized calcium carbonate from cockle shells. Briefly, cockle shells were boiled for 30 min in a steel container and thoroughly washed to remove stains on the shells. Upon drying in an oven (FD 115, Fisher Scientific, Limburg, Germany) at 50 °C, the shells were finely ground and then sieved using a stainless-steel laboratory test sieve (Endicott Ltd., London, UK) with an aperture size of 75 μm to obtain micro-sized calcium carbonate powder. 

For the preparation of CaCO_3_ NPs, 5 g of micro-sized calcium carbonate powder was added into 50 mL of deionized water in a 250 mL flat-bottom flask. The mixture was stirred with a magnetic stirrer bar at 1000 rpm for 30 min at room temperature using a magnetic stirring machine (WiseStir^®^ Systematic Multi-Hotplate Stirrer, Daihan Scientific^®^, Wonju, Republic of Korea), followed by adding 1.5 mL of BS-12 and stirring at 1000 rpm for 2 h at room temperature. The obtained samples were then centrifuged (Beckman Coulter, CA, USA) three times to remove BS-12 and dried in an oven at 50 °C for 5 d. The dried samples were placed in a glass cylindrical jar containing ceramic balls and rolled on a programmable ball miller (BML-6″, Daihan Scientific^®^, Republic of Korea) at 200 rpm for 500 h to obtain the final CaCO_3_ NPs.

### 2.3. Synthesis and Optimization of TQ-CaCO_3_ NPs

TQ-CaCO_3_ NPs were synthesized using two methods: a shaking incubator and a high-speed homogenizer. Briefly, different weight ratios of TQ to CaCO_3_ NPs (1:2, 1:4, 1:6, 1:8, 1:10, 1:15, and 1:20 *w*/*w*) were prepared. For the synthesis method using a shaking incubator, the mixtures (12 mg of CaCO_3_ NPs and varying amounts of TQ in 1.2 mL of ethanol) were continuously shaken at 200 rpm overnight in the dark at room temperature in the shaking incubator (LSI 3016R, Daihan Labtech, Wonju, Republic of Korea). For the synthesis method using a high-speed homogenizer (IKA-T25 digital Ultra-Turrax, Staufen, Germany), the mixtures (150 mg of CaCO_3_ NPs and varying amounts of TQ in 15 mL of ethanol) were homogenized at the time and speed-determined via single-factor experiments. The single-factor experimental design is shown in Supplementary Table S1. Finally, TQ-CaCO_3_ NPs were collected by centrifuging, washing, and drying and kept at room temperature for further use.

The supernatant of each formulated mixture was collected for compound loading content (LC) and encapsulation efficiency (EE) analyses. The better synthesis method and the best formulation were determined by comparing the results of LC and EE. The amount of free compound in the supernatant was evaluated using an ultraviolet–visible (UV-Vis) spectrophotometer (PerkinElmer Lambda 35, Perkin Elmer, Shelton, CT, USA), where the absorption peak of TQ in ethanol was 252 nm (Supplementary Figure S1). The results of LC and EE were determined by calculating the difference between the total weight of the compound fed and the free compound weight in the supernatant according to the following equations:

LC (%) = (weight of compound fed − weight of free compound)/weight of nanoparticles × 100%,

EE (%) = (weight of compound fed − weight of free compound)/weight of compound fed × 100%.

### 2.4. Physicochemical Characterization of CaCO_3_ NPs and TQ-CaCO_3_ NPs

#### 2.4.1. Electron Microscopy and Zetasizer 

The surface morphology of CaCO_3_ NPs and TQ-CaCO_3_ NPs was analyzed via field emission scanning electron microscopy (FESEM, Nova^TM^NanoSEM 230, FEI, Hillsboro, OR, USA) and high-resolution transmission electron microscopy (HRTEM, Hitachi H-7100, Tokyo, Japan). The nanoparticles were suspended in 100% acetone, followed by sonication with high-level ultrasonic power (Power Sonic 505, Seoul, Republic of Korea) for 15 min at room temperature. Subsequently, a drop of the dispersion was placed on a carbon-coated copper grid, and the samples were dried at room temperature before microscopy viewing. The size of nanoparticles in the HRTEM figure was measured using ImageJ 1.51n software. The zeta potential and size of nanoparticles were analyzed using Zetasizer Nano ZS (Malvern Instruments, Worcestershire, UK) at 25 °C. The dried nanoparticles were dispersed in 100% acetone for hydrodynamic size analysis and in deionized water (pH 7.4) for zeta potential analysis, where the Henry model was employed for zeta potential determination.

#### 2.4.2. Energy-Dispersive X-ray Spectroscopy, X-ray Diffractometer, and Fourier Transform Infrared Spectroscopy

Elemental compositions of CaCO_3_ NPs and TQ-CaCO_3_ NPs were determined using energy-dispersive X-ray spectroscopy (EDX, X-mas EDS, Oxford Instruments, Abingdon, UK), which is conjugated with FESEM. The crystalline nature and purity of CaCO_3_ NPs and TQ-CaCO_3_ NPs were determined using a Shimadzu X-ray diffraction (XRD)-6000 powder diffractometer on a continuous scanning process with Cu Kα (λ = 1.5406 Å) as the X-ray source. The obtained XRD data were analyzed using Jade 6 software, and the crystallographic model was created using Vesta 3.5.8 software. Crystallite sizes of CaCO_3_ NPs and TQ-CaCO_3_ NPs were obtained using Scherrer’s equation:

D = 0.9λ/B × cosθ,

where D is crystallite size in angstroms, λ is X-ray wavelength (1.5406 Å), B is full-width at half maximum of peak in radian unit, and θ is peak position in radian unit. The chemical properties of CaCO_3_ NPs and TQ-CaCO_3_ NPs were determined using Fourier transform infrared spectroscopy (FTIR, Spectrum 100, Perkin Elmer, Shelton, CT, USA) over the range of 4000 to 400 cm^−1^ at a 2 cm^−1^ resolution and averaging 64 scans. The obtained FTIR data were analyzed using OMNIC 7.3 software.

### 2.5. In Vitro Compound Release Study

The compound release study of TQ was monitored in PBS buffer with 1% (*v*/*v*) Tween 80 at pH 7.4, 6.5, and 5.0 using the pre-treated dialysis tubing cellulose membrane (Molecular weight cut-off: 14 kDa; Sigma-Aldrich). The dialysis was conducted in a shaking incubator at 37 ± 0.5 °C and 100 rpm. At predetermined time intervals, the release medium was withdrawn and replaced with the same volume of fresh medium. The quantity of TQ released from TQ-CaCO_3_ NPs was detected by UV-vis spectrophotometry. The compound release data were fitted in different kinetic models, including zero-order, first-order, Higuchi, Hixson–Crowell, and Krosmeyer–Peppas models. The R^2^ value was used to evaluate the best fit of the data to the kinetic models. The compound release mechanism was analyzed using the n value from the Krosmeyer–Peppas model.

### 2.6. Biocompatibility Evaluation of CaCO_3_ NPs and TQ-CaCO_3_ NPs

#### 2.6.1. Hemocompatibility Analysis

The rat red blood cells (erythrocytes) were collected from plasma by centrifugation at 3000 rpm for hemocompatibility analysis. The erythrocytes were redispersed in the PBS to prepare a 4% solution. The dispersed blood was added to each test sample aliquot at different concentrations (62.5–4000 μg/mL), followed by incubation for 1 h at 37 °C. Thereafter, the test samples were recentrifuged, and the supernatant was analyzed at 540 nm by microplate reader. The PBS solution and deionized water were used as negative (0% hemolysis) and positive control (100% hemolysis), respectively. The hemolysis percentage was calculated using the following equation:

hemolysis (%) = [(A_sample_ − A_negative_)/(A_positive_ − A_negative_)] × 100%,

where A_sample_, A_negative_, and A_positive_ represent the absorbance of the test sample, negative control, and positive control, respectively.

#### 2.6.2. Toxicity Analysis in Brine Shrimp

The toxicity was determined by measuring the survival rate of brine shrimp treated with different concentrations of nanoparticles [30]. Briefly, 34 g of sea salt (without iodine) was dissolved in 1 L of deionized water as the simulated seawater, and 1 g of brine shrimp eggs was put into the salt water. The eggs were then incubated with constant air and a light source (12 h light–dark cycle) at room temperature for 24 h. Once hatching, 10 active nauplii in 1 mL of salt water were transferred to each well of the 24-well plate. Different concentrations (0–1000 μg/mL) of CaCO_3_ NPs and TQ-CaCO_3_ NPs were added into the wells containing the nauplii, whereas KMnO_4_ dissolved in simulated seawater with the same concentrations served as the positive control. The surviving nauplii were observed using light microscopy (LM) and recorded at 12, 24, 36, and 48 h. The brine shrimp survival rate was calculated using the following formula:

survival rate (%) = alive brine shrimp/total brine shrimp × 100%.

#### 2.6.3. Cytotoxicity Analysis

Cytotoxicity of nanoparticles was evaluated in the NIH3T3 embryonic fibroblast cell line. NIH3T3 cells were cultured with different concentrations of CaCO_3_ NPs (concentrations: 0–1000 μg/mL), TQ (TQ concentrations: 0–60 μM), and TQ-CaCO_3_ NPs (TQ concentrations: 0–60 μM) for 96 h. After 96 h of incubation, 20 μL of MTT solution (5 mg/mL) was added to each well and incubated at 37 °C (5% CO_2_ and 95% air) for 4 h. The solution was then removed, and the purple formazan crystals were solubilized in DMSO. The absorbance was measured at 570 nm with reference wavelength of 630 nm using the multimode microplate reader (Synergy H1, BioTek, Winooski, VT, USA). The cell viability was determined with respect to the untreated control. Half-maximum inhibitory concentration (IC_50_) was calculated by nonlinear regression analysis in GraphPad Prism 8 software.

### 2.7. In Vitro Cell Experiments of TQ-CaCO_3_ NPs in Combination with 5-FU

#### 2.7.1. MTT Assay

Colon cancer CT26 cells (5 × 10^3^ cells/well) were seeded in 96-well plates for 24 h and treated with different concentrations of TQ-CaCO_3_ NPs, 5-FU, or in combination for 96 h. After treatment, the cells were incubated with MTT (5 mg/mL) reagent for 4 h. The purple formazan crystals formed were solubilized with DMSO, and the absorbance (OD) at 570 nm (630 nm as the reference wavelength) was measured using the multimode microplate reader (Synergy H1, BioTek, USA). The cell viability was determined with respect to the untreated control. IC_50_ was calculated by nonlinear regression analysis in GraphPad Prism 8 software. 

#### 2.7.2. Synergism Analysis

The combination index (CI) was calculated using CompuSyn 1.0 software, as established by Chou and Talalay, where CI > 1 indicates antagonism, CI = 1 indicates additive, and CI < 1 indicates synergism [31].

#### 2.7.3. Colony-Formation Assay

After being cultured in 6-well plates for 48 h, CT26 cells were treated with TQ-CaCO_3_ NPs, 5-FU, and their combination. After treatment, the cells were allowed to form colonies for 7 days until the colonies were visible. They were then fixed with 100% methanol and stained with 0.5% crystal violet. The cells were photographed using a microscope attached to a digital camera. The number of colonies was calculated using ImageJ software.

#### 2.7.4. AO/PI Staining

Following 96 h of treatment with TQ-CaCO_3_ NPs and 5-FU either alone or in combination, CT26 cells were washed with PBS and stained with 5 μg/mL AO/PI (1:1) dual stain for 10 min at room temperature. The cells were photographed using an inverted fluorescence microscope (Carl Zeiss Microscopy GmbH, Gottingen, Germany). AO-viable cell density was calculated using ImageJ software according to the presence or absence of non-viable cell staining (PI).

#### 2.7.5. Cell Cycle and Apoptosis Analysis

CT26 cells were cultured in 6-well plates with TQ-CaCO_3_ NPs and 5-FU either alone or in combination for 96 h. For cell cycle analysis, after being fixed in 70% ethanol overnight at 4 °C, cells were resuspended in PBS and exposed to RNase A solution (10 mg/mL) and PI solution (50 μg/mL) in the dark for 30 min. For apoptosis analysis, cells were resuspended in 1× binding buffer and stained with 5 μL of Annexin V-FITC and 5 μL of PI in the dark for 20 min. Thereafter, cell cycle and apoptosis were measured by a BD FACSCanto II flow cytometer (BD Biosciences, Milpitas, CA, USA) and analyzed using BD FACSDiva 6.1.2 software.

#### 2.7.6. Wound-Healing Assay

CT26 cells were seeded in 6-well plates and scraped with a 200 μL sterile pipette tip when the cells achieved 90% confluence. The scratched cells were then cultured with TQ-CaCO_3_ NPs and 5-FU either alone or in combination for 48 h. Images were captured using an inverted fluorescence microscope (Carl Zeiss Microscopy GmbH, Gottingen, Germany) after 0, 24, and 48 h. The wound-healing area was measured and analyzed using ImageJ software.

#### 2.7.7. The 3D Spheroid Culture

CT26 cells were seeded in agarose-coated 96-well plates at 2000, 4000, 6000, 8000, 10,000, 15,000, and 20,000 cells/well and incubated for 13 days at 37 °C. Spheroids were observed and imaged using a LM on days 4, 7, 10, and 13. Image J software was used to measure the diameter of spheroids and determine the volume of spheroids.

#### 2.7.8. The 3D Spheroid-Inhibition Assay

CT26 cell seeding density was chosen for obtaining the spheroids with a diameter of 400–500 μM after 4-day incubation. Spheroids were then cultured with TQ-CaCO_3_ NPs and 5-FU either alone or in combination for 96 h and imaged for a period of 14 days using an LM. The diameter and volume of spheroids were determined using Image J software. The spheroid growth inhibition rate was calculated using the following formula:

growth inhibition rate on the *n*th day (%) = (1 − Vt*n*/Vc*n*) × 100%,

where Vt*n* represents the spheroid volume of the treatment group on the *n*th day relative to its average spheroid volume on the 4th day, and Vc*n* is the average spheroid volume of the control group on the *n*th day relative to its average volume on the 4th day.

### 2.8. Network Pharmacology

#### 2.8.1. Target Prediction and Screening of 5-FU and TQ

The molecular structures of 5-FU and TQ were obtained from the PubChem database (https://pubchem.ncbi.nlm.nih.gov/, accessed on 16 February 2024). The biological targets of 5-FU and TQ were retrieved from the TCMSP (https://www.tcmsp-e.com, accessed on 16 February 2024), Swiss Target Prediction (http://www.swisstargetprediction.ch/, accessed on 16 February 2024), Stitch (http://stitch.embl.de/, accessed on 16 February 2024), TargetNet (http://targetnet.scbdd.com/, accessed on 16 February 2024), SuperPred (https://prediction.charite.de/, accessed on 16 February 2024), DrugBank (https://go.drugbank.com/, accessed on 16 February 2024), and SEA (https://sea.bkslab.org/, accessed on 16 February 2024) databases. Drug–target networks were constructed using Gephi 0.10.1 software.

#### 2.8.2. Targets Collection and Screening of Colon Cancer

“Colon cancer” was used as the keyword to retrieve the target genes from DisGeNET (https://www.disgenet.org/search, accessed on 17 February 2024), GeneCards (https://www.genecards.org/, accessed on 17 February 2024), OMIM (https://www.omim.org/, accessed on 17 February 2024), PharmGKB (https://www.pharmgkb.org/, accessed on 17 February 2024), and TTD (https://db.idrblab.net/ttd/, accessed on 17 February 2024) databases. Meanwhile, with “colon cancer” as the keyword and “Homo sapiens” as the target organism, the GSE74602 dataset was selected from GEO (https://www.ncbi.nlm.nih.gov/geo/, accessed on 18 February 2024) database, including 30 paired normal and tumor samples. The “limma” package in R 4.2.2 software was used to screen the differential genes with *p* value < 0.05 and |logFC| > 1. The volcano map and heat map were drawn using R 4.2.2 software for visualization of differential genes. After consolidating the target genes and eliminating the duplicate values, the final targets of colon cancer were obtained. 

#### 2.8.3. PPI, GO, and KEGG Analysis

The potential targets of 5-FU in combination with TQ against colon cancer were identified by intersecting the target genes of 5-FU, TQ, and colon cancer using Origin 9.8.0.200 software, and a Venn diagram was drawn for illustration. STRING database (https://string-db.org/, accessed on 19 February 2024) was used to construct the PPI network of the drug–colon cancer intersection targets. Gephi 0.10.1 software was applied to perform data analysis and visualize the PPI network. Based on the degree values, the node size and color of targets were adjusted, and the hub genes were screened. GO functional annotation and KEGG pathway analysis of the drug–colon cancer targets after STRING processing was conducted by using the “clusterProfiler”, “org.Hs.eg.db”, and “ggplot2” packages in R 4.2.2 software. The top 10 GO terms with the smallest *p*-value were selected and are shown in a bar graph and chord diagram, and the top 30 KEGG pathways with the smallest *p*-value are shown in a bubble graph. Simultaneously, the top 5 pathways with the smallest *p*-value and colorectal cancer pathway obtained by enrichment analysis of KEGG and their corresponding targets were imported into Gephi 0.10.1 software to draw the key pathway–target network diagram.

### 2.9. Molecule Docking

The 3D structures of 5-FU and TQ were downloaded from the PubChem database (https://pubchem.ncbi.nlm.nih.gov/, accessed on 22 February 2024), and the protein structures of the target genes were obtained from the PDB database (https://www.rcsb.org/, accessed on 22 February 2024). Discovery Studio Visualizer v19.1.0.18287 software was used to remove water molecules of proteins, predict the binding sites of protein pockets, and delete the original ligands. The processed proteins were imported into AutoDock Tools 1.5.6 software to add polar hydrogen and charge. The molecular docking was executed using AutoDock Vina 1.2.5 software. Subsequently, the results of molecular docking were visualized in Discovery Studio Visualizer v19.1.0.18287 software.

### 2.10. Statistical Analysis

Data analysis was performed with at least triplicates using the GraphPad Prism 8 and Origin 9.8.0.200 software. Statistical analysis involved one-way ANOVA followed by Turkey’s post-test for multiple comparisons or Student’s *t*-test for single comparison and two-way ANOVA followed by Dunnett’s post-test. *p* value < 0.05 (*p* < 0.05) was considered statistically significant. Unless otherwise stated, the results were expressed as mean ± standard deviation.

## 3. Results

### 3.1. Synthesis of the Optimal TQ-CaCO_3_ NPs

**Figure 1:**
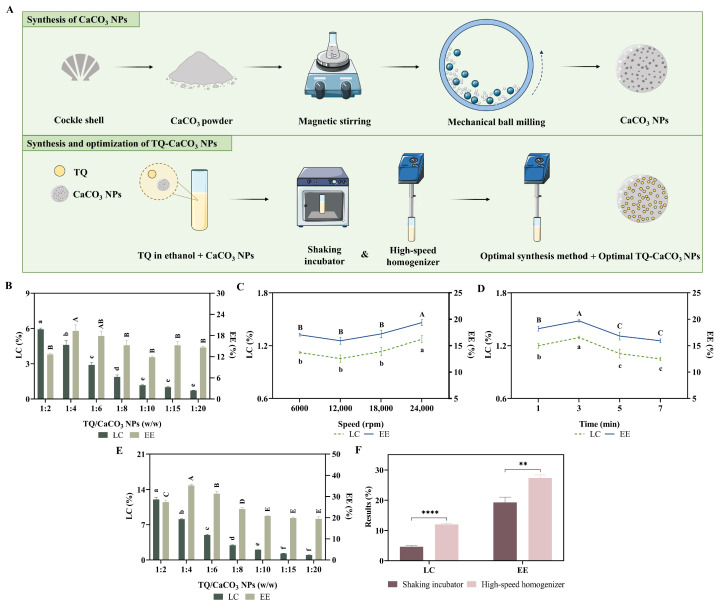
(**A**) Schematic illustration of CaCO_3_ NPs and TQ-CaCO_3_ NPs synthesis; (**B**) LC and EE of TQ in TQ-CaCO_3_ NPs at varying weight ratios between TQ and CaCO_3_ NPs, using a shaking incubator for compound loading; (**C**) LC and EE of TQ in TQ-CaCO_3_ NPs at varying speeds in the single-factor experiment; (**D**) LC and EE of TQ in TQ-CaCO_3_ NPs at varying times in the single-factor experiment; (**E**) LC and EE of TQ in TQ-CaCO_3_ NPs at varying weight ratios between TQ and CaCO_3_ NPs, using a high-speed homogenizer for compound loading; (**F**) comparison of optimized loading results obtained using a shaking incubator and a high-speed homogenizer. The results with the different superscripts (a–f and A–E nomenclature) indicate significant differences (*p* < 0.05). ** *p* < 0.01, **** *p* < 0.0001. LC, loading content; EE, encapsulation efficiency.

The synthesis concept of CaCO_3_ NPs and TQ-CaCO_3_ NPs is illustrated in Figure 1A. The most critical parameters for loading the compound into nanoparticles are the weight percentage of the compound in the delivery system (LC) and the percentage of the encapsulated compound to its initial amount for loading (EE). Optimizing EE can reduce compound loss by minimizing the amount of left compound in the loading process, and optimal LC ensures the dose of the encapsulated compound before administration and minimizes the amount of nanocarrier, thereby reducing the potential side effects of nanocarrier [32]. Firstly, TQ loading at varying weight ratios (weight of TQ to weight of CaCO_3_ NPs) was performed using the shaking incubator to obtain the best formulation. Figure 1B showed that the highest EE in the 1:4 group (19.31%) had no significant difference from the result of the 1:6 group (17.88%). However, the LC in the 1:4 group was significantly superior to that in the 1:6 group (*p* < 0.05). Therefore, when the shaking incubator was used for TQ loading, the 1:4 weight ratio of TQ to CaCO_3_ NPs was considered the optimal formulation.

To increase the interactions between the compounds and nanoparticles for better loading results, a high-speed homogenizer with strong thrust forces is considered a potentially effective method for compound loading. Two factors will affect the loading results when the high-speed homogenizer is utilized for compound loading: speed and time [33]. Thus, the single-factor experiments were performed to determine the optimal speed and time for TQ loading. As the results illustrate in Figure 1C,D, loading with 24,000 rpm and 3 min achieved the highest LC and EE of TQ-CaCO_3_ NPs in the single-factor experiments, respectively, employed as the target speed and time for TQ loading using the high-speed homogenizer. Subsequently, TQ loading at varying weight ratios of TQ to CaCO_3_ NPs with the target speed and time was tested for the best formulation. Although it is displayed in Figure 1E that loading at a 1:4 weight ratio reached the highest EE of TQ-CaCO_3_ NPs with 8.82% of LC, the LC of loading at a 1:2 weight ratio (13.68%) was significantly superior to that of other groups (*p* < 0.05). In addition, loading at a 1:2 weight ratio still obtained high EE (27.36%). Therefore, the 1:2 weight ratio of TQ to CaCO_3_ NPs was deemed the optimal formulation. Moreover, the optimal outcomes achieved using the high-speed homogenizer for TQ loading surpassed those attained using the shaking incubator (*p* < 0.05), as shown in Figure 1F. Overall, the high-speed homogenizer was chosen as the final synthesis method for TQ-CaCO_3_ NPs, where the 1:2 weight ratio of TQ to CaCO_3_ NPs at a speed of 24,000 rpm for 3 min was employed for loading TQ.

### 3.2. Characterization of CaCO_3_ NPs and TQ-CaCO_3_ NPs

**Figure 2:**
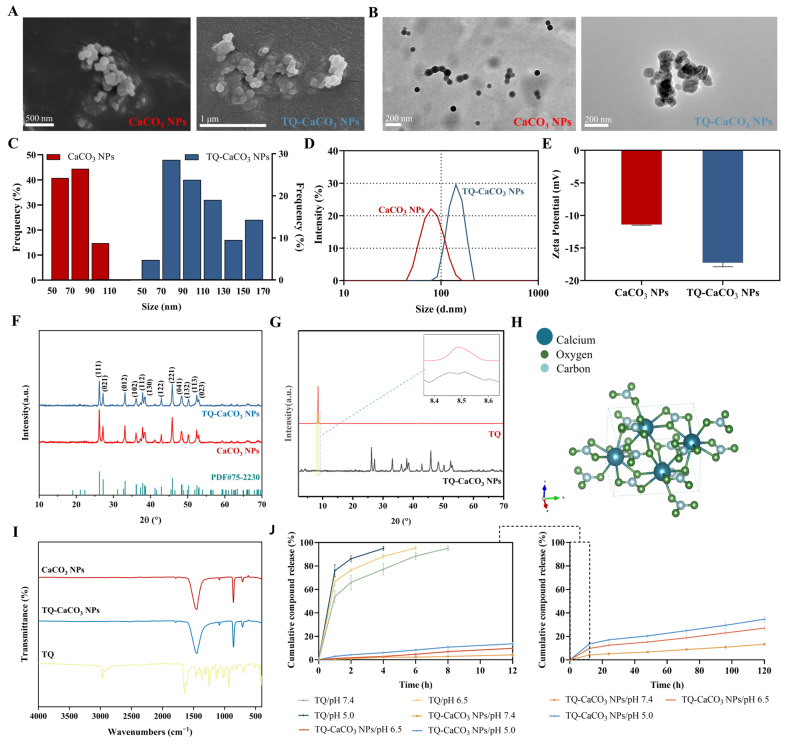
Surface morphology of CaCO_3_ NPs and TQ-CaCO_3_ NPs in (**A**) FESEM and (**B**) HRTEM; (**C**) particle size distribution of CaCO_3_ NPs and TQ-CaCO_3_ NPs in HRTEM; (**D**) hydrodynamic size and (**E**) zeta potential of CaCO_3_ NPs and TQ-CaCO_3_ NPs; (**F**) XRD patterns of CaCO_3_ NPs, TQ-CaCO_3_ NPs, and the reference diffraction peak (PDF#75-2230) of aragonite calcium carbonate nanoparticles from Inorganic Crystal Structure Database. Those labeled are the Miller indices planes (characteristic peaks of aragonite phase); (**G**) XRD pattern comparison between TQ and TQ-CaCO_3_ NPs; (**H**) orthorhombic crystalline morphology of aragonite CaCO_3_ NPs; (**I**) FTIR spectra of CaCO_3_ NPs, TQ-CaCO_3_ NPs, and TQ. (**J**) in vitro compound release profiles of TQ-CaCO_3_ NPs and TQ in PBS with 1% (*v*/*v*) Tween 80 at 37 °C and pH 7.4, 6.5, and 5.0. FESEM, field emission scanning electron microscopy; HRTEM, high-resolution transmission electron microscopy; XRD, X-ray diffraction; FTIR, Fourier transform infrared spectroscopy; PBS, phosphate-buffered saline.

Both FESEM and HRTEM images of CaCO_3_ NPs and TQ-CaCO_3_ NPs display homogeneous spherical or nearly spherical porous nanoparticles (Figure 2A,B). Meanwhile, HRTEM images show an average nanoparticle size of 77 nm before compound loading and 110 nm after loading (Figure 2C). Additionally, the hydrodynamic size distribution and zeta potential of CaCO_3_ NPs and TQ-CaCO_3_ NPs are illustrated in Figure 2D,E, respectively. Dynamic light scattering revealed consistent results in the hydrodynamic diameter of CaCO_3_ NPs and TQ-CaCO_3_ NPs with the results in HRTEM, where the average hydrodynamic diameter of CaCO_3_ NPs and TQ-CaCO_3_ NPs was 86 and 139 nm, respectively, with a polydispersity index of less than 0.5. The zeta potential of CaCO_3_ NPs was −11.4 mV, determined by electrophoretic light scattering, whereas TQ-CaCO_3_ NPs had a slightly higher zeta potential of −17.3 mV. Moreover, the elemental compositions of synthesized CaCO_3_ NPs and TQ-CaCO_3_ NPs were determined using EDX analysis (Supplementary Figures S2–S4). As displayed in Table 1, CaCO_3_ NPs contained 16.06% carbon, 62.42% oxygen, 21.27% calcium, and 0.25% sodium, suggesting that calcium carbonate derived from the cockle shell was almost 100% pure. Based on the conventional stoichiometrically derived ratio of CaCO_3_, the Ca/C/O was almost in a molar ratio of 1:1:3. The EDX results of TQ-CaCO_3_ NPs and TQ suggested that the change in elemental percentages of Ca/C/O between CaCO_3_ NPs and TQ-CaCO_3_ NPs may be due to the loading of the compound. 

XRD was used to analyze the crystalline phase of CaCO_3_ NPs before and after TQ loading. The CaCO_3_ NP diffraction peaks (crystal planes) observed, i.e., 26.22° (111), 27.23° (021), 33.15° (012), 36.12° (102), 37.90° (112), 38.42° (130), 42.92° (122), 45.87° (221), 48.33° (041), 50.25° (132), 52.48° (113), and 53.05° (023), confirmed the aragonite structure of CaCO_3_ NPs when comparing them with the reference diffraction peaks (PDF#75-2230) of aragonite calcium carbonate nanoparticles from the Inorganic Crystal Structure Database (Figure 2F). The similarity in the diffraction peaks of CaCO_3_ NPs and TQ-CaCO_3_ NPs indicated that the aragonite structure of CaCO_3_ NPs remained unchanged even after loading with TQ. In addition, the similar diffraction peak between TQ and TQ-CaCO_3_ NPs shown in Figure 2G suggests that TQ has been loaded. Moreover, the lattice parameters of CaCO_3_ NPs and TQ-CaCO_3_ NPs are displayed in Table 2. Notably, the crystallite size of CaCO_3_ NPs and TQ-CaCO_3_ NPs was 827 and 848 Å, respectively, consistent with the results in HRTEM and Zetasizer, and the crystallinity of both nanoparticles was more than 95%. The changes in lattice parameters between CaCO_3_ NPs and TQ-CaCO_3_ NPs further demonstrated that TQ has been loaded. Based on the obtained lattice parameters, the orthorhombic (Pmcn space group) crystalline morphology of aragonite CaCO_3_ NPs was created and illustrated in Figure 2H.

FTIR was used to detect the functional groups of nanoparticles and analyze their chemical properties. The FTIR spectra of CaCO_3_ NPs, TQ-CaCO_3_ NPs, and TQ are shown in Figure 2I, and the peak vibration assignments of nanoparticles are presented in Table 3. CaCO_3_ NPs exhibited characteristic peaks at 707.41, 854.8, and 1082.21 cm^−1^, corresponding to in-plane C-O bending vibration, out-of-plane C-O bending vibration, and symmetric C-O stretching of CO_3_^2-^ in aragonite polymorph, respectively. This suggests that CaCO_3_ NPs were an aragonite crystal, in agreement with the result of XRD analysis. The prominent peak for the asymmetric C-O stretching of CO_3_^2-^ appeared at 1450.53 cm^−1^, and the weak peak at 1783.63 cm^−1^ was attributed to symmetric C-O stretching and in-plane C-O bending vibration of CO_3_^2-^. It is interesting to know that TQ showed its major characteristic peaks for CH (sp_3_), CH (sp_2_), C꞊C, and trans C꞊C groups at 2962.28, 3256.94, 1639.50, and 931.67 cm^−1^, respectively. The FTIR absorption peaks of TQ-CaCO_3_ NPs were similar to those of aragonite CaCO_3_ NPs, implying that the aragonite polymorphism did not change after TQ loading. However, the broader absorbance band in TQ-CaCO_3_ NPs around 1447.33 cm^−1^, compared to that in CaCO_3_ NPs, and the shift of peaks, such as from 1450.53 cm^−1^ in CaCO_3_ NPs to 1447.33 cm^−1^ in TQ-CaCO_3_ NPs, can be attributed to the addition of TQ to CaCO_3_ NPs.

The compound-release study was investigated at a pH of 7.4, 6.5, and 5.0, which modeled the blood and healthy tissue environment, the weakly acidic extracellular environment in malignant tumors, and the more acidic intracellular environment in malignant tumors, respectively [34]. Figure 2J displays the TQ release profiles at pH 7.4, 6.5, and 5.0 for 120 h. As evident in the figure, the release of TQ from TQ-CaCO_3_ NPs was pH-dependent. The accumulative TQ release amounts of TQ-CaCO_3_ NPs at pH 7.4 were 5.27% within 24 h and 13.30% within 120 h. The release rate at pH 6.5 was higher than that at pH 7.4, with the accumulative release amounts 12.50% within 24 h and 27.01% within 120 h. At pH 5.0, the TQ release rate was accelerated more obviously. TQ-CaCO_3_ NPs released 17.94% of TQ within 24 h and 33.77% within 120 h at pH 5.0. These results suggested the pH-sensitive property of CaCO_3_ NPs and its potent benefits in increasing the intracellular accumulation of TQ in tumors, which may strengthen the anticancer effects of TQ. The compound-release pattern of TQ-CaCO_3_ NPs was analyzed using zero-order, first-order, Higuchi, Hixon–Crowell, and Korsmeyer–Peppas models. It can be observed in Table 4 that the release curves of TQ-CaCO_3_ NPs at pHs of 7.4 and 5.0 exhibited the best fit with the Korsmeyer–Peppas model (R^2^ = 0.98578 at a pH of 7.4 and R^2^ = 0.98765 at a pH of 5.0). Higuchi was the best-fit model for TQ-CaCO_3_ NPs release at a pH of 6.5 (R^2^ = 0.98363), indicating the concentration of TQ released from TQ-CaCO_3_ NPs increased with the square root of time [35]. However, the high R^2^ (0.98266) suggested that the compound-release pattern of TQ-CaCO_3_ NPs at a pH of 6.5 also followed the Korsmeyer–Peppas model. Furthermore, TQ-CaCO_3_ NPs released TQ following the non-Fickian transport mechanism (0.45 < n < 0.89) [36]. Therefore, the hydrophobic compound TQ was released from TQ-CaCO_3_ NPs with both diffusion and erosion-controlled principals.

### 3.3. CaCO_3_ NPs and TQ-CaCO_3_ NPs Exhibit Favorable Biocompatibility

**Figure 3:**
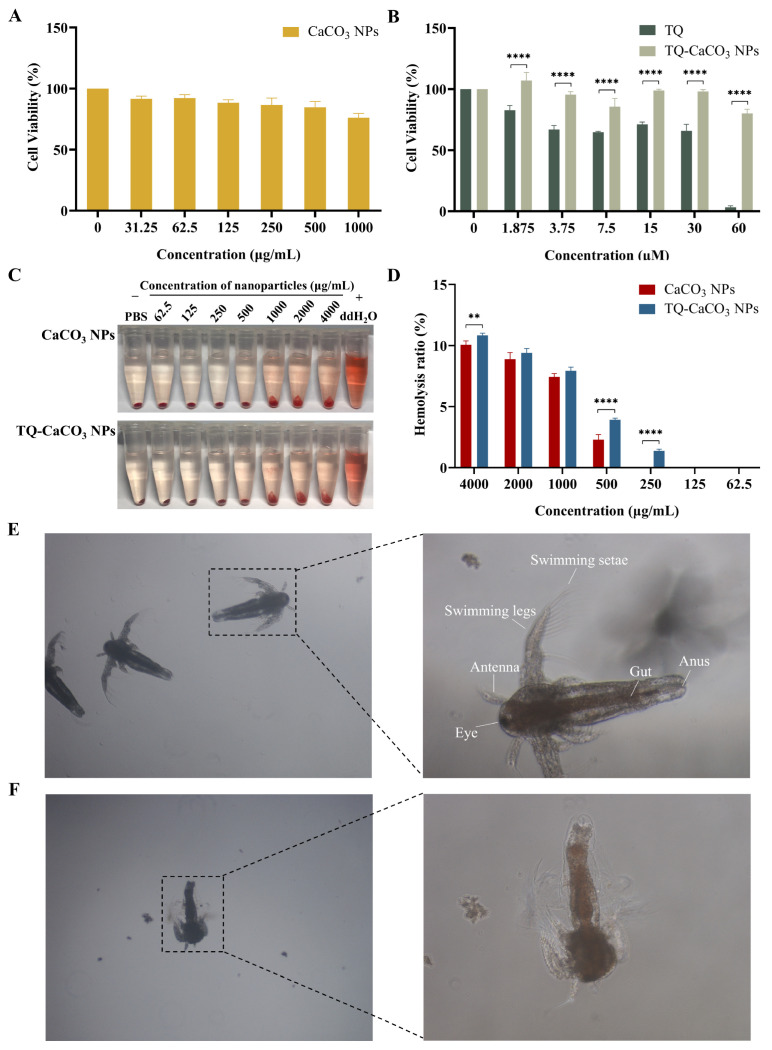
Cytotoxicity of (**A**) CaCO_3_ NPs (concentrations: 0–1000 μg/mL), as well as (**B**) TQ and TQ-CaCO_3_ NPs (TQ concentrations: 0–60 μM) in NIH3T3 embryonic fibroblast cell line for 96 h; (**C**) hemolysis phenomena and (**D**) hemolysis ratios of CaCO_3_ NPs and TQ-CaCO_3_ NPs. PBS solution and ddH_2_O were used as the negative (0% hemolysis) and positive control (100% hemolysis), respectively. The solution with red color means the release of hemoglobin from the damaged erythrocytes, and the red pellet at the bottom of the tube is intact erythrocytes after centrifugation; (**E**) morphology of live brine shrimp at 4× and 10× magnifications; (**F**) morphology of dead brine shrimp at 4× and 10× magnifications. ** *p* < 0.01, **** *p* < 0.0001. PBS, phosphate-buffered saline; ddH_2_O, deionized water.

Although calcium carbonate nanoparticles are well-known for excellent biocompatibility and calcium carbonate has been approved by the FDA for clinical application, it is still necessary to evaluate the biocompatibility (cytotoxicity, toxicity, and blood compatibility) of nanoparticles due to the potential problems in the process of nanoparticle preparation. To investigate the cytotoxicity of nanoparticles, the normal embryonic fibroblast cell line NIH3T3 was employed as the cell model, and an extended incubation time of 96 h was chosen. According to the standard ISO 10993-5, non-toxicity is considered the reduction of cell viability by no more than 30% [37]. As shown in Figure 3A, blank CaCO_3_ NPs exhibited cell viability of more than 75% within the concentration range of 0–1000 μg/mL after 96 h, suggesting they are non-toxic to NIH3T3 cells. Figure 3B reveals that TQ was toxic to NIH3T3 cells, with an IC_50_ value of 21.08 ± 2.49 μM, but TQ-CaCO_3_ NPs were non-toxic to NIH3T3 cells even at the high TQ concentration of 60 μM. This may be attributed to the prolonged release of TQ from CaCO_3_ NPs under the healthy cell environment of pH 7.4. Therefore, CaCO_3_ NP loading of TQ can reduce the toxicity of TQ.

The blood compatibility of CaCO_3_ NPs and TQ-CaCO_3_ NPs was evaluated by the in vitro erythrocyte-induced hemolysis test. This test assessed the release percentage of hemoglobin when nanoparticles were directly in contact with erythrocyte surfaces and caused cell lysis, regarded as the preliminary investigation for the in vivo toxicity evaluation of nanoparticles [38]. During this test, isotonic PBS solution was used as the negative control, and the positive control was deionized water that can induce osmotic shock, leading to hemolysis [39]. Figure 3C displays the results of the hemolysis test, revealing that the hemolysis of CaCO_3_ NPs and TQ-CaCO_3_ NPs was concentration-dependent. At the same time, complete lysis (the absence of precipitation) was observed in deionized water-treated erythrocytes. Generally, more than 25% of the hemolysis ratio is considered hemolytic, and less than 10% is non-hemolytic [40]. It can be observed in Figure 3D that CaCO_3_ NPs and TQ-CaCO_3_ NPs exhibited low hemolysis rates (below 12%) even at the high concentration of 4000 μg/mL. Remarkably, the hemolysis ratio of CaCO_3_ NPs was better than that of TQ-CaCO_3_ NPs, especially at concentrations of 500 and 250 μg/mL (*p* < 0.05), which may be associated with the effects of TQ on erythrocytes. Moreover, the two samples with concentrations ranging from 62.5 to 500 μg/mL showed less than 5% hemolysis activity. Therefore, it can be concluded that the synthesized CaCO_3_ NPs and TQ-CaCO_3_ NPs possessed great blood biocompatibility.

Brine shrimp (Artemia salina), an invertebrate species belonging to the zooplankton, was used to evaluate the toxicity of nanoparticles due to its advantages of a fast life cycle, low maintenance costs, and adaptability to laboratory conditions [41,42]. Based on the brine shrimp survival results after treatment of positive control KMnO_4_, KMnO_4_ exhibited toxicity to brine shrimp, with an LC_50_ (lethal concentration 50) value of 433.07 ± 77.00, 306.73 ± 18.91, and 110.23 ± 4.35 μg/mL for 24, 36, and 48 h of exposure, respectively. According to Clarkson’s toxicity index, KMnO_4_ was non-toxic (LC_50_ value more than 1000 μg/mL) for 12 h but moderately toxic (LC_50_ value between 500 and 100 μg/mL) after further extended exposure to 24, 36, and 48 h [43]. In contrast, the brine shrimp toxicity tests of CaCO_3_ NPs and TQ-CaCO_3_ NPs did not show any sign of toxicity, as no dead brine shrimp was recorded in varying concentrations of nanoparticles even for 48 h of incubation, indicating that CaCO_3_ NPs and TQ-CaCO_3_ NPs were non-toxic. Moreover, the morphology of brine shrimp was observed using the LM. Figure 3E displays the live brine shrimp with an eye, swimming legs, swimming setae, gut, and anus, whereas the dead brine shrimp with a damaged body is shown in Figure 3F. Based on these findings and considering the results of blood compatibility evaluation and cytotoxicity analysis, the synthesized CaCO_3_ NPs were safe for drug delivery in the body, and TQ-CaCO_3_ NPs exhibited excellent biocompatibility.

### 3.4. TQ-CaCO_3_ NPs in Combination with 5-FU Inhibits the Proliferation of CT26 Cells

**Figure 4:**
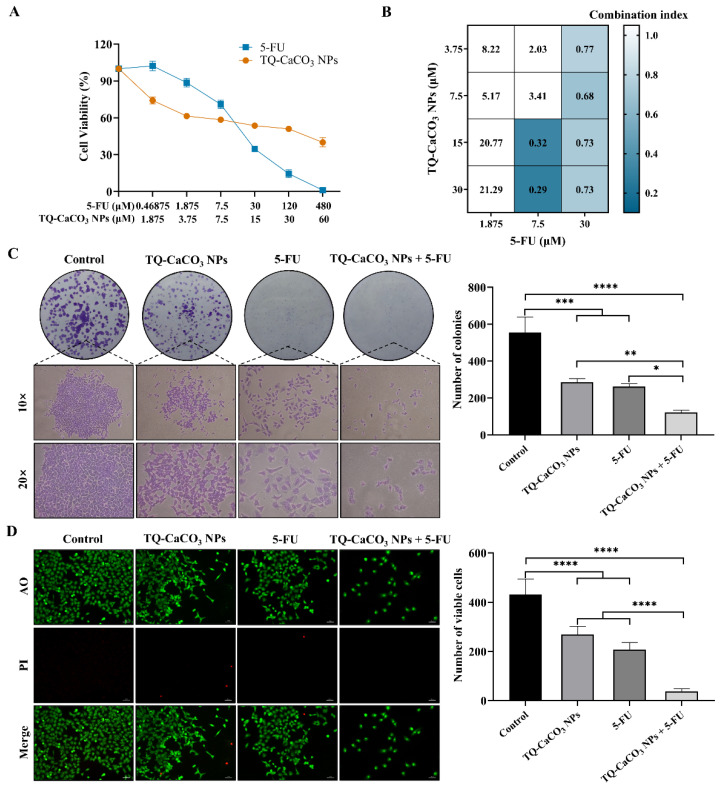
(**A**) Cytotoxicity of TQ-CaCO_3_ NPs (TQ concentrations: 0–60 μM) and 5-FU (concentrations: 0–480 μM) on CT26 cells for 96 h; (**B**) combination index heat map of TQ-CaCO_3_ NPs and 5-FU in combination against CT26 cells; (**C**) colony-formation assay of CT26 cells treated with TQ-CaCO_3_ NPs (15 μM) and 5-FU (7.5 μM) alone or in combination, with the represented images under 10× and 20× magnification; (**D**) AO/PI staining assay of CT26 cells treated with TQ-CaCO_3_ NPs (15 μM) and 5-FU (7.5 μM) alone or in combination, with the represented images under 20× magnification, where viable cells in green by AO staining and non-viable cells in red due to PI staining. * *p* < 0.05, ** *p* < 0.01, *** *p* < 0.001, **** *p* < 0.0001.

To explore the effects of TQ-CaCO_3_ NPs in combination with 5-FU against colon cancer, an MTT assay was performed to determine the cell viability in colon cancer CT26 cells. As shown in Figure 4A, both TQ-CaCO_3_ NPs and 5-FU exhibited concentration-dependent inhibitive effects on the cell viability of CT26 cells, with the IC_50_ values of TQ-CaCO_3_ NPs and 5-FU being 23.35 and 17.23 μM, respectively. On this basis, the inhibitory effect of TQ-CaCO_3_ NPs (3.75, 7.5, 15, and 30 μM) combined with 5-FU (1.875, 7.5, and 30 μM) on CT26 cells was further evaluated. Compared to the individual treatments, the increased inhibition of cell viability was observed in half of the combination groups, while the remaining combination groups reduced the interaction effect on CT26 cells. The CI value of every TQ-CaCO_3_ NPs and 5-FU combination was calculated to elucidate the synergistic effects of the two treatments. The results showed that the CI values of 7.5 μM of 5-FU combined with TQ-CaCO_3_ NPs (15 and 30 μM) and 30 μM of 5-FU combined with TQ-CaCO_3_ NPs (3.75–30 μM) were all less than 1, indicating there was a synergistic effect between TQ-CaCO_3_ NPs and 5-FU (Figure 4B). Among all synergistic groups, 30 μM of TQ-CaCO_3_ NPs combined with 7.5 μM of 5-FU had the lowest CI value, suggesting the strongest synergistic effect, followed by 15 μM of TQ-CaCO_3_ NPs combined with 7.5 μM of 5-FU. However, considering that a smaller dose can achieve similar effects, 15 μM of TQ-CaCO_3_ NPs combined with 7.5 μM of 5-FU was selected to further analyze its inhibitive effects on colon cancer. 

The colony-formation results showed that TQ-CaCO_3_ NPs (15 μM) and 5-FU (7.5 μM) inhibited the colony formation of CT26 cells, with an average of 286 and 262 colonies, respectively, compared with the average of 555 colonies in the control group (Figure 4C). In addition, as shown in Figure 4D, the reduced viable cells after the treatment of TQ-CaCO_3_ NPs (15 μM) and 5-FU (7.5 μM) were observed in the AO/PI staining results. Notably, consistent with the results in the MTT assay, the colony-formation assay and AO/PI-staining assay revealed the synergism of TQ-CaCO_3_ NPs (15 μM) and 5-FU (7.5 μM), where the combination of TQ-CaCO_3_ NPs and 5-FU significantly suppressed the colony formation and growth of CT26 cells. Overall, these findings imply the synergistic inhibitive effects between TQ-CaCO_3_ NPs and 5-FU on cell proliferation in colon cancer.

### 3.5. TQ-CaCO_3_ NPs in Combination with 5-FU Induces Cell Cycle Arrest and Apoptosis in CT26 Cells

**Figure 5:**
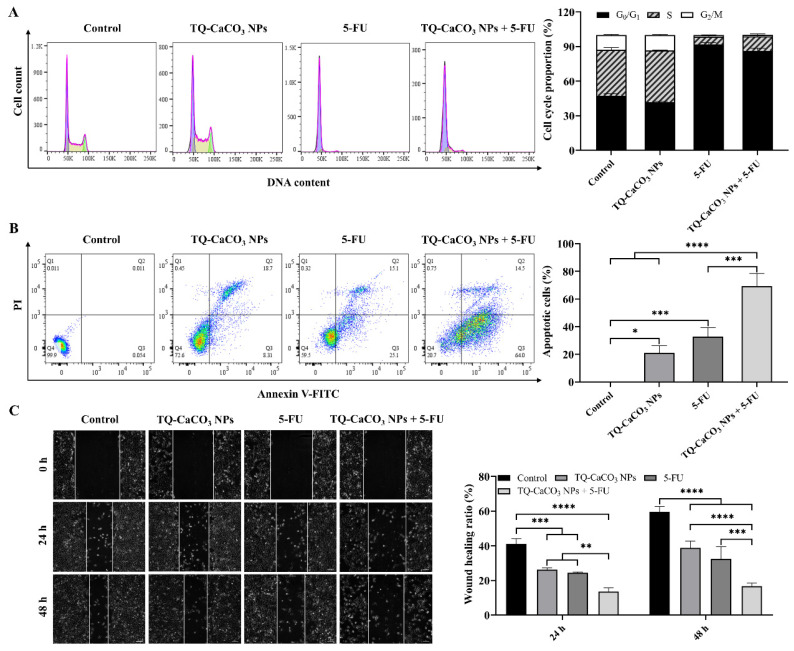
(**A**) Cell cycle assay of CT26 cells treated with TQ-CaCO_3_ NPs (15 μM) and 5-FU (7.5 μM) alone or in combination, with the represented images displaying cell cycle distributions; (**B**) Annexin V-FITC assay of CT26 cells treated with TQ-CaCO_3_ NPs (15 μM) and 5-FU (7.5 μM) alone or in combination, with the represented images depicting cells in different quadrants: necrosis (Q1), late apoptosis (Q2), early apoptosis (Q3), and live (Q4), where Q2 and Q3 are collectively called apoptotic cells; (**C**) wound-healing assay of CT26 cells treated with TQ-CaCO_3_ NPs (15 μM) and 5-FU (7.5 μM) alone or in combination, with the represented images under 10× magnification showing cell migration after 24 and 48 h. * *p* < 0.05, ** *p* < 0.01, *** *p* < 0.001, **** *p* < 0.0001.

The effects of TQ-CaCO_3_ NPs in combination with 5-FU on cell cycle and apoptosis in CT26 cells were evaluated to investigate the underlying mechanism of TQ-CaCO_3_ NPs combined with 5-FU against colon cancer. As illustrated in the flow cytometry results in Figure 5A, cells treated with 5-FU or TQ-CaCO_3_ NPs combined with 5-FU prominently increased G_0_/G_1_ phase arrest and reduced the cell number in the S and G_2_/M phases. However, after TQ-CaCO_3_ NPs treatment, the proportion of cells in the S phase increased slightly, while that in the G_0_/G_1_ phase decreased slightly, and that in the G_2_/M phase had no obvious change, indicating that TQ-CaCO_3_ NPs may arrest the cell cycle in the S phase. Additionally, treatment with TQ-CaCO_3_ NPs or 5-FU alone resulted in the significant apoptosis of CT26 cells (*p* < 0.05) (Figure 5B). The co-treatment of TQ-CaCO_3_ NPs and 5-FU showed a significant increase in the apoptotic cells (69.40%), including 54.17% with early apoptosis and 15.23% with late apoptosis, compared with the individual treatments (*p* < 0.05). In conclusion, the combination of TQ-CaCO_3_ NPs (15 μM) and 5-FU (7.5 μM) exerted synergistic antiproliferative effects on CT26 cells by inducing cell cycle arrest and apoptosis, ultimately causing cell death.

### 3.6. TQ-CaCO_3_ NPs in Combination with 5-FU Inhibits the Migration of CT26 Cells

A wound-healing assay was performed to investigate the potential effects of TQ-CaCO_3_ NPs in combination with 5-FU on the CT26 cell migration rate. As displayed in Figure 5C, both TQ-CaCO_3_ NPs and 5-FU exhibited a potent migration-inhibiting efficiency on CT26 cells. Nonetheless, the inhibitory effects of TQ-CaCO_3_ NPs combined with 5-FU were more noticeable than TQ-CaCO_3_ NPs or 5-FU alone, as the wound closure was distinctly disrupted. These results suggested that TQ-CaCO_3_ NPs (15 μM) and 5-FU (7.5 μM) synergistically inhibited the migration of CT26 cells.

### 3.7. TQ-CaCO_3_ NPs in Combination with 5-FU Suppresses the Growth of CT26 Spheroids

**Figure 6:**
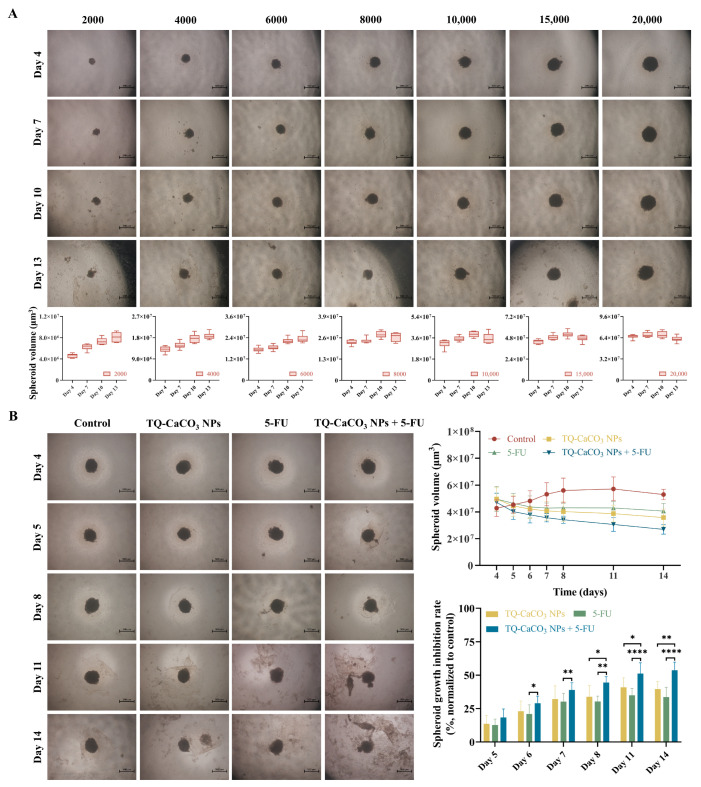
(**A**) CT26 spheroid growth analysis for culturing for 13 days, with the represented images under 4× magnification showing that spheroid size and growth trend depend on the initial cell seeding density (2000 to 20,000 cells/well); (**B**) growth-inhibition analysis of CT26 spheroids treated with TQ-CaCO_3_ NPs (15 μM) and 5-FU (7.5 μM) alone or in combination, with the represented images under 4× magnification displaying the spheroid volume and growth inhibition. * *p* < 0.05, ** *p* < 0.01, **** *p* < 0.0001.

Compared with conventional 2D cells, 3D spheroids provide a tissue structure similar to intact human tumors, consisting of concentric layers including a proliferating zone, quiescent zone, and necrotic core [44]. Different cell seeding densities were applied to monitor the size of CT26 spheroids. Figure 6A revealed that the size of spheroids was strongly dependent on the initial cell seeding density. CT26 spheroids formed at the initial cell seeding density of 15,000 cells/well and cultured for 4 days had a diameter of 400–500 μm, conforming to the concentrically layered structure, thus serving as the model for spheroid-inhibition analysis [45]. After spheroid formation on day 4, CT26 spheroids were treated with TQ-CaCO_3_ NPs (15 μM) and 5-FU (7.5 μM) alone or in combination for 96 h. While untreated spheroids increased in size, both TQ-CaCO_3_ NPs and 5-FU exhibited inhibitory effects on spheroid growth (Figure 6B). Moreover, cell proliferation was significantly inhibited after incubation with the combination of TQ-CaCO_3_ NPs and 5-FU, and the spheroids were severely destroyed on day 14 (*p* < 0.05). These data indicated that TQ-CaCO_3_ NPs combined with 5-FU could significantly suppress CT26 spheroid growth.

### 3.8. Network Pharmacology

#### 3.8.1. Potential Targets of Drugs and Colon Cancer

**Figure 7:**
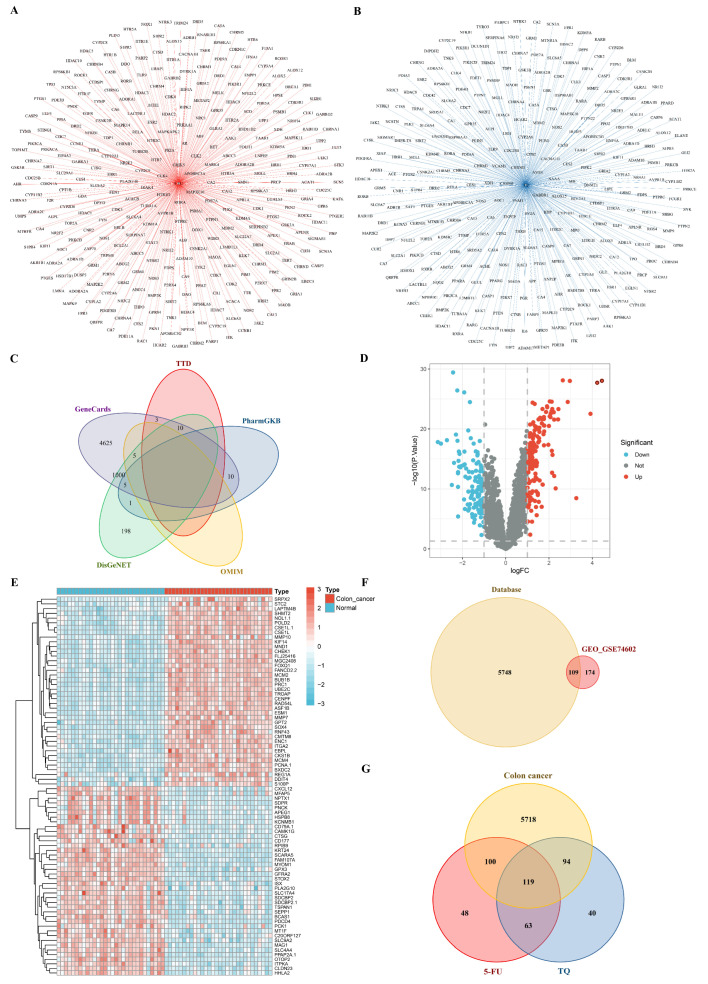
(**A**) 5-FU-target network; (**B**) TQ-target network; (**C**) Venn diagram of colon cancer-related targets from the 5 databases; (**D**) volcano map of all differential genes, with red dots for up-regulated genes and blue dots for down-regulated genes; (**E**) heat map of top 40 differential genes; (**F**) Venn diagram of colon cancer-related targets from the 5 databases and GSE74602 dataset; (**G**) Venn diagram of 5-FU, TQ, and colon cancer-related targets, with a total of 119 intersection genes.

After being obtained from the TCMSP, Swiss Target Prediction, Stitch, TargetNet, SuperPred, DrugBank, and SEA databases, the target genes were standardized using the uniport database, and duplicate genes were excluded. 5-FU and TQ contained 330 and 316 potential targets, respectively, with the drug–target network diagrams shown in Figure 7A,B. Meanwhile, 1220, 5658, 5, 16, and 13 colon cancer-associated genes were screened from DisGeNET, GeneCards, OMIM, PharmGKB, and TTD databases, respectively. A total of 5857 targets related to colon cancer were obtained after merging and deduplicating the genes of the five databases (Figure 7C). Moreover, the differential analysis results of the GSE74602 dataset revealed that 283 genes expressed differentially between normal samples and colon tumor samples, including 153 up-regulated genes and 130 down-regulated genes (Figure 7D,E). Subsequently, the target genes from five databases and the differential genes from the GSE74602 dataset were consolidated into 6031 genes related to colon cancer. Finally, 119 targets of 5-FU combined with TQ for colon cancer treatment were screened, as illustrated in the Venn diagram (Figure 7G).

#### 3.8.2. PPI Network and GO Functional Annotation

**Figure 8:**
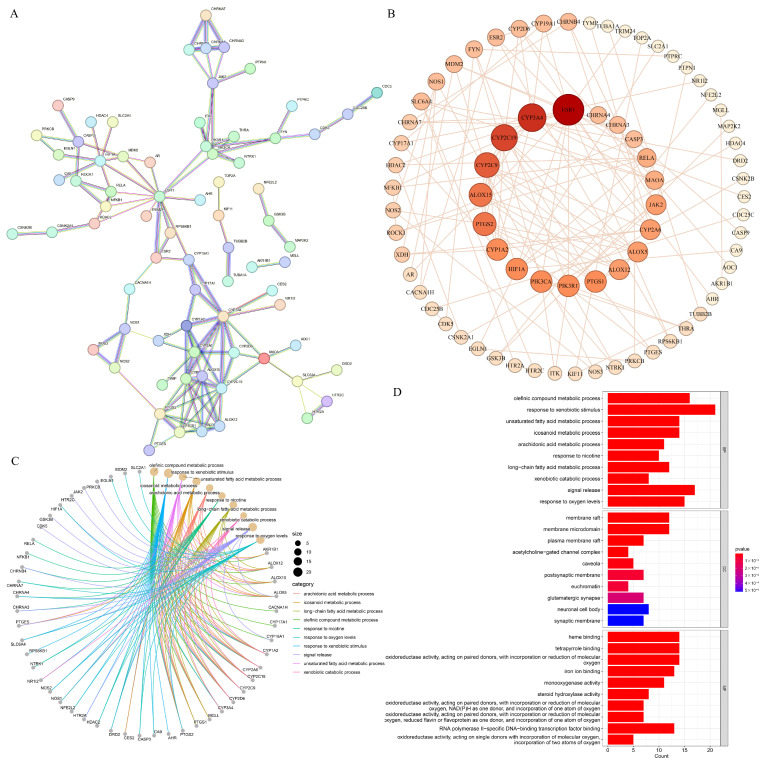
(**A**) PPI network of the 74 core genes in STRING database; (**B**) PPI network of the 74 genes, where the deeper the color and the bigger the size, the more significant the target is for colon cancer treatment by 5-FU in combination with TQ; (**C**) the top 10 BPs with corresponding genes, where the size of the dot in the diagram represents the number of genes; (**D**) GO functional annotation of the 74 genes: the top 10 entries of BP, CC, and MF. BP, biological process; CC, cellular component; MF, molecular function.

The 119 intersection genes of 5-FU in combination with TQ against colon cancer were imported into the STRING database, where the interaction score was set greater than 0.9, determining 74 core targets and protein interaction relationships (Figure 8A). The PPI network of the 74 targets was further analyzed using Gephi 0.10.1 software, and the network diagram is shown in Figure 8B with 74 nodes and 250 edges. The deeper the color of the nodes and the bigger the size of the nodes, the greater the degree value and the more edges connected to the target, indicating the higher significance of the target in the network. *ESR1*, *CYP3A4*, *CYP2C19*, *CYP2C9*, and *ALOX15* were the top five hub genes. The GO functional annotation included 1028 biological processes (BPs), 41 cellular components (CCs), and 111 molecular functions (MFs) (*p* < 0.05). The top enrichment entries of BP involved in 5-FU combined with TQ against colon cancer are the response to xenobiotic stimulus, signal release, and the olefinic compound metabolic process, with the corresponding targets displayed in Figure 8C; the CCs were mainly enriched in the membrane raft, membrane microdomain, and neuronal cell body; the top three MFs were related to heme binding, tetrapyrrole binding, and oxidoreductase activity, acting on paired donors, with the incorporation or reduction in molecular oxygen (Figure 8D).

#### 3.8.3. KEGG Pathway Enrichment Analysis

**Figure 9:**
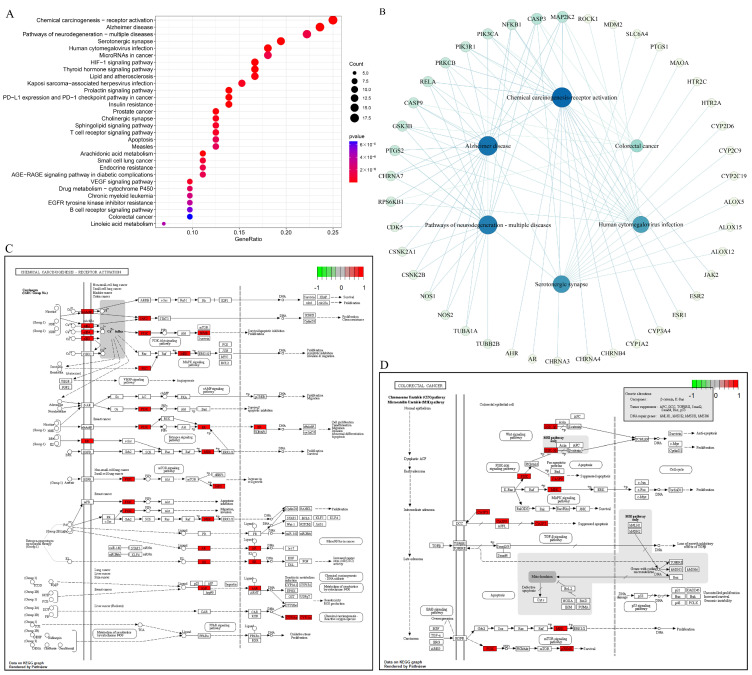
(**A**) KEGG pathway enrichment of the 74 core genes: the top 30 enriched pathways; (**B**) pathway–target network, where the deeper the color, the more genes from the 74 targets involved in the pathway; (**C**) chemical carcinogenesis-receptor activation pathway and (**D**) colorectal cancer pathway, where red marks represent the genes from the 74 targets.

The KEGG pathway enrichment analysis of the 74 core targets was performed to further investigate the potential molecular mechanisms of 5-FU in combination with TQ against colon cancer. Figure 9A shows that the KEGG pathways were mainly enriched in chemical carcinogenesis-receptor activation, Alzheimer’s disease, pathways of neurodegeneration-multiple disease, serotonergic synapse, and human cytomegalovirus infection. Remarkably, the colorectal cancer pathway was also in the top 30 KEGG pathways (*p* < 0.05). The pathway–target network (Figure 9B) was constructed to better depict the relationship between the targets and the key pathways (the top five pathways and the colorectal cancer pathway). Meanwhile, the pathway–target network revealed that the top five hub genes in terms of degree value were *MAP2K2*, *CASP3*, *NFKB1*, *PIK3CA*, and *PIK3R1*. Moreover, the top chemical carcinogenesis-receptor activation pathway and colorectal cancer pathway are visualized in Figure 9C,D, respectively.

### 3.9. Molecular Docking

**Figure 10:**
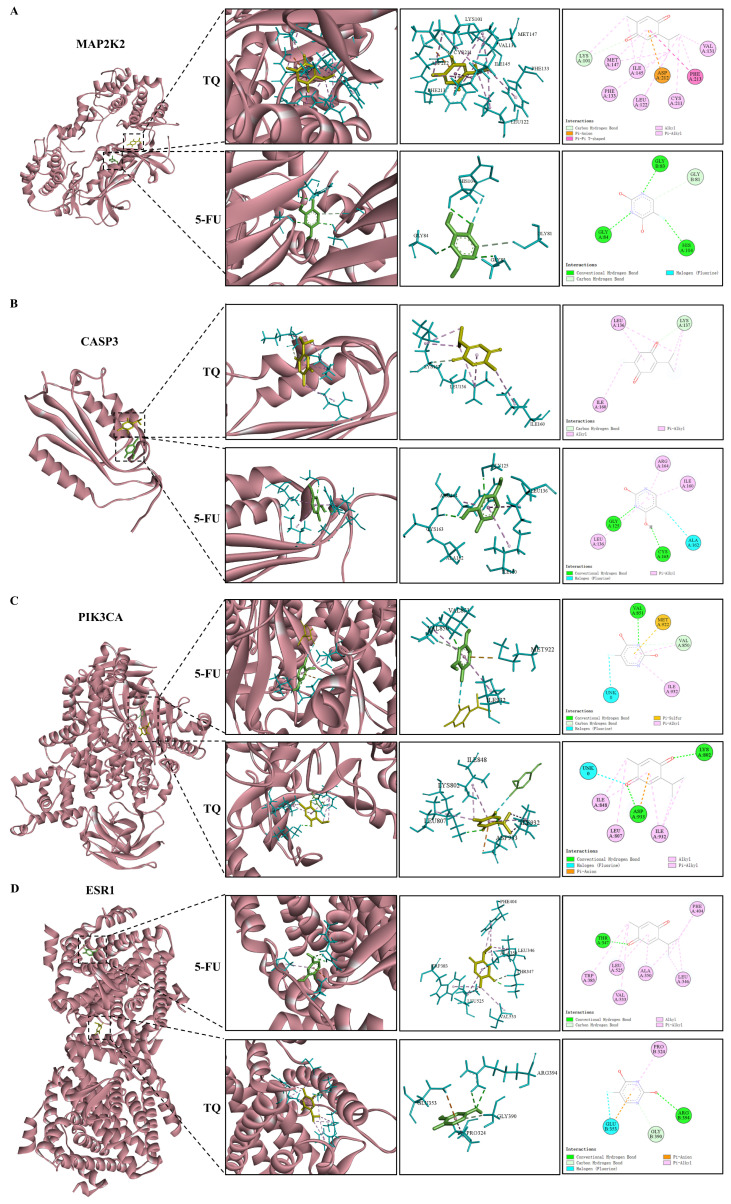

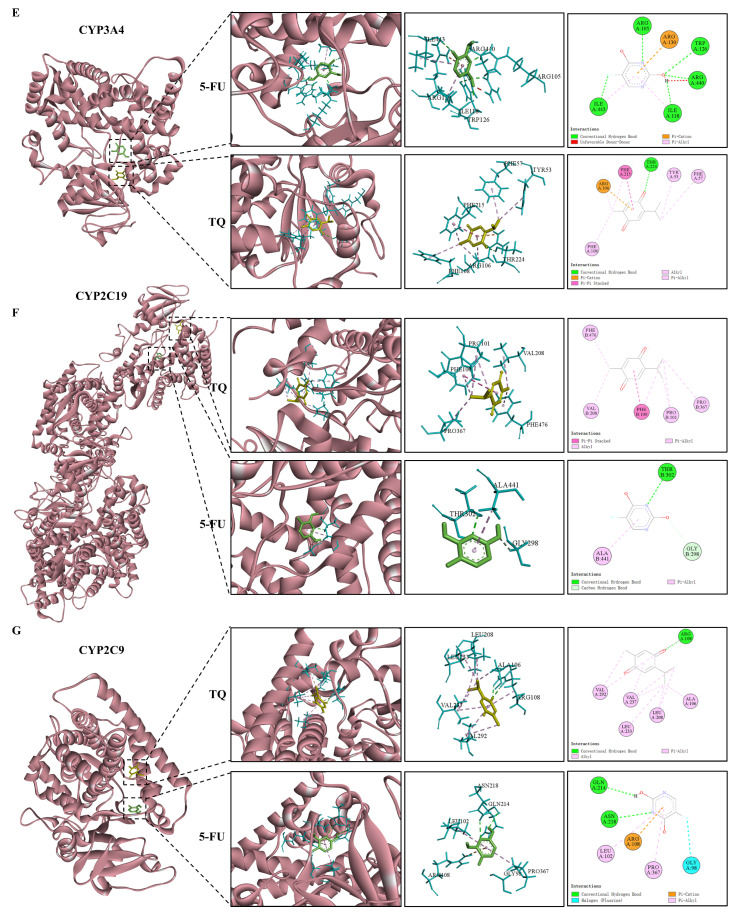
Schematic diagram of molecular docking between 5-FU and TQ and (**A**) *MAP2K2*, (**B**) *CASP3*, (**C**) *PIK3CA*, (**D**) *ESR1*, (**E**) *CYP3A4*, (**F**) *CYP2C19*, as well as (**G**) *CYP2C9*.

The hub genes were screened from the PPI network of the 74 core targets and pathway–target network of the six key pathways, where *MAP2K2*, *CASP3*, *PIK3CA*, *ESR1*, *CYP3A4*, *CYP2C19*, and *CYP2C9* were used to conduct molecular docking with 5-FU and TQ. The molecular docking results in Table 5 indicate that all targets had favorable binding activities with 5-FU and TQ. Then, 2D force and 3D spatial environment images were employed to further analyze the interactions between 5-FU and TQ and the hub genes, as shown in Figure 10. Among them, 5-FU formed a hydrogen bond interaction with *PIK3CA* at the residue of VAL-851, a pi–sulfur bond interaction at the residue of MET-922, a carbon–hydrogen bond interaction at the residue of VAL-850, and a pi–alkyl bond interaction at the residue of ILE-932. Meanwhile, TQ interacted with *PIK3CA* by forming a hydrogen bond at the residues of ASP-933 and LYS-802, a pi–anion bond at the residue of ASP-933, and alkyl and pi–alkyl bonds at the residues of ILE-848, LEU-807 as well as ILE-932. It is noted that 5-FU and TQ also interacted (halogen (fluorine) bond) when binding to *PIK3CA*. Overall, the anti-colon cancer effects of 5-FU in combination with TQ and its potential therapeutic targets were verified from the molecular docking level.

## 4. Discussion

Despite medical advances in cancer treatment, colon cancer remains a common cause of cancer-related death worldwide. As a first-line chemotherapeutic drug for colon cancer therapy, 5-FU inhibits thymidylate synthase and incorporates its metabolites into RNA and DNA, leading to the death of cancer cells [46]. However, nearly half of colon cancer are resistant to 5-FU-based chemotherapy, and a high dose of 5-FU may provoke severe side effects [47,48]. Therefore, the combinatorial use of 5-FU and phytochemical compounds may be an alternative to reduce these risks due to different mechanisms of action and reduced dosage. The natural antioxidant TQ has been reported to help maintain cellular homeostasis and prevent the progression of various oxidative stress-related diseases, including cancer, cardiovascular diseases, and neurodegenerative disorders [49,50,51,52]. Notably, TQ, in combination with 5-FU, has shown promising anticancer properties in the treatment of triple-negative breast and gastric cancers [9,10]. In addition, owing to its antioxidant properties, TQ mitigates common side effects such as mucositis, myelosuppression, and gastrointestinal toxicity associated with 5-FU treatment [53]. TQ also shows hepatoprotective and renal protective properties against drug cytotoxicity, affecting key enzymes involved in detoxification, such as aspartate transaminase and alanine transaminase [54]. Nonetheless, considering that the hydrophobicity and poor bioavailability of TQ are barriers to its clinical translation and that nano delivery of phytochemical compound is a novel and effective targeted anticancer approach, this study combined TQ-loaded calcium carbonate nanoparticles (TQ-CaCO_3_ NPs) with 5-FU for the treatment of colon cancer.

In the present study, porous cockle shell-derived CaCO_3_ NPs with particle sizes of approximately 80 nm were developed using a facile mechanical ball-milling method to deliver compound TQ. This was the first attempt wherein different loading techniques, i.e., shaking incubator and high-speed homogenizer, were explored to prepare the optimal TQ-CaCO_3_ NPs. The slightly negative surface charge of CaCO_3_ may facilitate weak electrostatic attractions with TQ, particularly through its polar functional groups. The shaking incubator and high-speed homogenizer used during the compound loading process enhanced these interactions by increasing the contact between TQ molecules and the CaCO_3_ surface. Given the hydrophobic nature of TQ, it is also likely that hydrophobic interactions play a significant role in the compound loading process, potentially occurring at less polar regions of the CaCO_3_ surface or within nanoparticle pores. Moreover, the use of a shaking incubator and high-speed homogenizer improved the interaction between TQ and CaCO_3_ by increasing collision frequency and mixing efficiency. This mechanical energy likely promoted the adsorption of TQ onto the nanoparticle surface through electrostatic and hydrophobic interactions. However, TQ loading using a high-speed homogenizer showed superior LC and EE values compared to loading with a shaking incubator, possibly because loading using a shaking incubator is a simple shaking method, whereas loading using a high-speed homogenizer with strong physical forces is more efficient [55]. The high-speed homogenizer rapidly promotes the interactions between compounds and nanoparticles by intense mechanical forces, leading to faster compound loading and improved loading results compared with gentler agitation provided by the shaking incubator. On the other hand, high-speed homogenization facilitates a uniform dispersion of compounds and prevents nanoparticle aggregation, contributing to a consistent and reproducible compound-loading process [56]. Therefore, the high-speed homogenizer was used as the compound loading method, and the optimal formulation (1:2 weight ratio of TQ to CaCO_3_ NPs at 24,000 rpm for 3 min) was employed for TQ loading.

Remarkably, CaCO_3_ NPs loading of the hydrophobic antioxidant TQ in the present study showed better loading results than CaCO_3_ NPs loading of the hydrophilic drug 5-FU in our previous study [57]. For one thing, this may be related to the solubility of compounds [32]. TQ in ethanol has better solubility than 5-FU in deionized water, which may contribute to a more homogenous distribution of TQ molecules in ethanol to promote better loading into CaCO_3_ NPs [58,59]. This also helps to explain why the best loading results of 5-FU were at a lower weight ratio, whereas TQ obtained the best loading results at a higher weight ratio. Conversely, the dispersity of CaCO_3_ NPs in the loading medium may be an important factor in compound loading [56]. CaCO_3_ NPs may disperse better in organic solvent ethanol than in water due to the stronger viscosity of ethanol, which may increase the surface area of CaCO_3_ NPs for compound loading, resulting in more effective interactions between TQ and CaCO_3_ NPs [60,61].

Due to the porosity of CaCO_3_ NPs, TQ molecules were incorporated within the pores of CaCO_3_ NPs during the high-speed mixing of TQ and CaCO_3_ NPs using a high-speed homogenizer. The size of TQ-CaCO_3_ NPs was slightly increased compared to the size of empty CaCO_3_ NPs, which may be attributed to the loading of TQ. Moreover, the particle sizes of CaCO_3_ NPs and TQ-CaCO_3_ NPs are suitable for compound delivery for cancer treatment, as nanoparticles with sizes between 20 and 150 nm can reduce liver clearance and kidney filtration and prolong the circulation time in vivo [62]. As reported, other elements except for Ca, C, and O have been found inside calcium carbonate nanoparticles synthesized by other methods, such as Si, Cu, P, and Mg [63,64,65]. In contrast, the synthesis method of calcium carbonate nanoparticles and compound-loading method in this study produced purer CaCO_3_ NPs and TQ-CaCO_3_ NPs. Moreover, other calcium carbonate phases have their characteristic peaks (crystal planes), such as the calcite phase at 29.32° (104), 35.90° (110), 39.36° (113), 47.47° (018), 48.43° (116), as well as 57.37° (122) and the vaterite phase at 20.89° (002), 24.82° (100), 26.99° (101), 32.72° (102), 43.71° (110), 49.07° (112), 50.06° (104), as well as 55.74° (202) [66]. However, these peaks do not appear in Figure 2F, indicating the synthesized CaCO_3_ NPs and TQ-CaCO_3_ NPs were pure aragonite. Notably, the characteristic diffraction peak of TQ was extremely weak in the XRD pattern of TQ-CaCO_3_ NPs, possibly associated with the encapsulation of TQ into the pores rather than the surface of CaCO_3_ NPs. Besides XRD, FTIR is another tool for comparing different calcium carbonate phases, where the calcite, vaterite, and aragonite phases exhibit their characteristic absorption bands. The results of FTIR validated the aragonite polymorphism of TQ-CaCO_3_ NPs and the encapsulation of TQ into CaCO_3_ NPs without chemical modification, as there was no new observed peak besides the characteristic peaks of CaCO_3_ NPs and TQ [67].

The release profile of compounds from nanoparticles is divided into a sudden release and a sustained release. The sudden release of compounds quickly reaches their effective therapeutic concentrations in the body, whereas sustained release keeps compounds within the effective therapeutic concentration range [68]. As illustrated in Figure 2J, TQ-CaCO_3_ NPs suddenly released nearly 6% within 2 h, while pure TQ rapidly released more than 95% within 8 h. Compared to the rapid release of pure TQ, the sustained release of TQ-CaCO_3_ NPs can maintain the effective concentrations of TQ, thus reducing the dose, frequency of administration, and adverse effects of the compound [69]. It is noted that the antioxidant TQ was released slower from TQ-CaCO_3_ NPs compared with the chemotherapy drug 5-FU from 5FU-CaCO_3_ NPs [57]. This could be related to the hydrophobic nature of TQ, encapsulated deeper inside the CaCO_3_ NPs, which had to follow a lengthier diffusion path to reach the surface in comparison to water-soluble 5-FU encapsulated near the surface [35]. Interestingly, pure 5-FU exhibited a similar rapid release profile at a pH of 7.4, 6.5, and 5.0, while pure TQ released faster at a lower pH value, which may be due to the hydrophilic nature of 5-FU and the increased solubility of TQ at the decreased pH value [12]. Furthermore, as demonstrated in cytotoxicity analysis, blood compatibility evaluation, and brine shrimp toxicity assessment, the synthesized CaCO_3_ NPs and TQ-CaCO_3_ NPs exerted excellent biocompatibility, showing promise for clinical application.

TQ-CaCO_3_ NPs, in combination with 5-FU, were then evaluated for their potency against colon cancer CT26 cells. Our results indicated that the combination of TQ-CaCO_3_ NPs and 5-FU inhibited the development and progression of colon cancer by regulating cell proliferation, apoptosis, and migration. Both TQ-CaCO_3_ NPs and 5-FU suppressed the viability of CT26 cells in a dose-dependent manner, whereas the antiproliferative effect of TQ-CaCO_3_ NPs in combination with 5-FU depended on their combined concentration. The synergism of two treatments can be inferred when the combination of the treatments at specific doses produces an effect greater than the sum of the effects achieved by the individual treatments at the same doses [70]. The CI values were calculated to measure the extent of drug interactions and revealed the optimal synergistic combination (CI = 0.32) of TQ-CaCO_3_ NPs and 5-FU against CT26 cells. In our previous study, the combinatorial effects of 5FU-CaCO_3_ NPs and TQ were investigated against colon cancer CT26 cells, which also showed synergism [57]. Notably, the optimal CI value of TQ-CaCO_3_ NPs and 5-FU was superior to that (0.60) of 5FU-CaCO_3_ NPs and TQ, indicating that TQ-CaCO_3_ NPs and 5-FU exhibited better synergistically inhibitory effects on CT26 cells. The colony-formation assay and AO/PI staining assay results further visually confirmed the synergistically inhibitive effects of the optimal combination between TQ-CaCO_3_ NPs and 5-FU on cell proliferation. Hence, we continued investigating the synergistic anti-colon cancer effects and the underlying therapeutic mechanisms of TQ-CaCO_3_ NPs (15 μM) and 5-FU (7.5 μM). 

As demonstrated, the combination of TQ-CaCO_3_ NPs and 5-FU exhibited antiproliferative activity by inducing cell cycle arrest and apoptosis in CT26 cells. 5-FU increased the G_0_/G_1_ phase arrest of CT26 cells and reduced the cell number in the S and G_2_/M phases, consistent with the mechanism of 5-FU, which inhibits thymidylate synthase, leading to the inhibition of DNA synthesis, induction of DNA damage, and a halt in cell cycle progression in the G1 or S phase [3]. In contrast, TQ-CaCO_3_ NPs slightly increased the cell number in the S phase and decreased in the G_0_/G_1_ phase. However, TQ-CaCO_3_ NPs and 5-FU combination prominently induced G_0_/G_1_ and S phases’ cell cycle arrest in CT26 cells, suggesting that TQ-CaCO_3_ NPs may enhance the efficacy of 5-FU by further reinforcing the cell cycle arrest in G_0_/G_1_ and S phases, resulting in a stronger inhibitory effect on cell proliferation. The enhancement of 5-FU inhibition of cell proliferation by TQ-CaCO_3_ NPs was further confirmed in the cell apoptosis assay. Cancer cell migration is an essential step for successful cancer metastasis associated with substantial mortality after surgical resection of primary cancer; thereby, inhibiting cell migration is a practical approach for anticancer therapy [71,72]. The wound-healing assay results revealed that TQ-CaCO_3_ NPs and 5-FU inhibited CT26 cell migration, while TQ-CaCO_3_ NPs combined with 5-FU exerted a more significant migration-inhibitory effect.

Before clinical trials, drugs must undergo a series of tests to ensure their effectiveness and safety, the first step of which is usually in vitro experiments based on 2D cells. However, compared with traditional 2D cells, 3D cell spheroids in vitro models with similar characteristics to solid tumors in vivo can better predict drug effects, bridging the gap between in vitro 2D cells and in vivo animal models [44]. In this study, agarose-based liquid overlay technology was applied to produce 3D spheroids, which has the advantage of allowing rapid aggregation of cells into spheroids with reproducible morphology [73]. Typically, when 3D spheroids are 400–500 μm in size, cell layers similar to those in solid tumors can be formed, including an outer layer composed of proliferating cells, a middle layer of quiescent cells, and a hypoxic inner layer of necrotic cells [45]. However, limited literature has explored the optimal initial cell seeding density for CT26 cells to form the expected spheroid size. To achieve a 3D spheroid size typical of solid tumors, we used increased cell seeding densities (2000, 4000, 6000, 8000, 10,000, 15,000, and 20,000 cells/well) to culture spheroids. It was observed that when the cell density was 15,000 cells/well, the spheroids cultured for 4 days were stable and uniform and reached the expected size, thereby being used for a spheroid-inhibition assay. As observed in Figure 6B, 5-FU had a less inhibitive effect on CT26 spheroids, possibly due to the spheroids exhibiting resistance to 5-FU. Nonetheless, the addition of TQ-CaCO_3_ NPs to 5-FU showed a high inhibitory effect on the spheroids, indicating that TQ-CaCO_3_ NPs enhanced the chemosensitivity of 5-FU to CT26 spheroids, thereby improving the therapeutic effect on colon cancer. Especially after 3 days of treatment, the inhibitory effect of 5-FU and TQ-CaCO_3_ NPs co-treatment was significantly better than that of the individual treatment (*p* < 0.05).

To the best of our knowledge, this is the first study to combine network pharmacology and molecular docking to elucidate the potential therapeutic mechanisms of 5-FU in combination with TQ (from TQ-CaCO_3_ NPs) against colon cancer. Network pharmacology, as a combination of system biology and network informatics, has been widely used as a useful analytical approach to predict potential pharmacological actions of drugs [74]. In this study, network pharmacology was employed to screen potential targets and analyze underlying therapeutic mechanisms of 5-FU in combination with TQ for the treatment of colon cancer. The targets of 5-FU and TQ and colon cancer-related genes were intersected, and 74 core targets of the 5-FU combined with TQ against colon cancer were screened. Meanwhile, the hub genes were identified in the PPI network of the 74 core targets based on the degree values, namely, *ESR1*, *CYP3A4*, *CYP2C19*, *CYP2C9*, and *ALOX15*. *ESR1* encoding estrogen receptor α expresses very low in normal colon mucosa, but an increase in *ESR1* expression results in poor outcomes in patients with colon cancer [75,76]. In addition, activated *ESR1* stimulates the expression of tumor promoters and promotes colon cancer cell survival and metastasis; thus, blocking *ESR1* expression offers a potential therapeutic opportunity for colon cancer [76]. Lipoxygenases and cytochrome P450s are the main enzymes mediating the metabolism of polyunsaturated fatty acids involved in cancer initiation, progression, and metastasis [77]. The expression of 15-lipoxygenase-1 (*ALOX15*), which plays a tumor-suppressing role, is reduced in human cancers, particularly colon cancer [78]. Transgenic expression of *ALOX15* suppresses TNF-α and iNOS expression as well as NF-κB activation and downregulates LRP5 to suppress Wnt/β-catenin signaling, thereby inhibiting colon cancer [79,80]. Moreover, previous studies have shown that *CYP3A4* was significantly downregulated in colonic tumors compared with normal mucosa tissues, whereas *CYP2C19* and *CYP2C9* were highly expressed in colon cancer; thus, upregulating *CYP3A4* expression and downregulating *CYP2C19* and *CYP2C9* expression may prohibit the growth and metastasis of colon cancer cells [81,82].

GO and KEGG analyses provide an in-depth description of the functions of potential therapeutic targets, reflecting the possible mechanisms of TQ in combination with 5-FU against colon cancer. Some important BPs, such as the response to xenobiotic stimulus, signal release, olefinic compound metabolic process, response to oxygen levels, and unsaturated fatty acid metabolic process, are related to the potential targets. KEGG enrichment results revealed that multiple signaling pathways are involved in the anti-colon cancer mechanisms of TQ combined with 5-FU, with six key pathways, i.e., colorectal cancer, chemical carcinogenesis-receptor activation, Alzheimer’s disease, neurodegeneration-multiple disease, serotonergic synapse, and human cytomegalovirus infection. Notably, five hub genes were identified based on the network between the six pathways and corresponding targets, including *MAP2K2*, *CASP3*, *NFKB1*, *PIK3CA*, and *PIK3R1*. *MAP2K2* (also known as *MEK2*), part of the Kras/Braf/MEK/MAPK signaling pathway, plays a vital role in inducing cell survival and proliferation and the inhibition of cell apoptosis of colon cancer [83]. The inhibition of *MAP2K2* may suppress not only the formation of colon cancer but also the progression of colon cancer and its metastasis to the liver and lung [84,85]. Activation of the PI3K-Akt-mTOR signaling pathway is essential to the survival, proliferation, migration, and invasion of various cancer cells [86]. In this signaling pathway, the oncogene *PIK3CA*, encoding the p110α catalytic subunit of PI3K, is one of the most commonly mutated genes in colon cancer [87,88]. In contrast, *PIK3R1* (p85α regulatory subunit of PI3K) is a tumor-suppressor gene, and the protein p85α has a reduced capacity to restrain p110α when *PIK3R1* is mutated, resulting in activation of PI3K-Akt-mTOR pathway [89]. Executioner *CASP3*, commonly used as a marker for the efficacy of cancer treatment, is involved in modulating colon cancer cell survival, proliferation, apoptosis, migration, invasion, and metastasis [90,91]. *NFKB1* (p50, p105, or p50/p105) is a member of the NF-κB signaling pathway. As activation of the NF-κB pathway promotes the formation and development of colon cancer through inducing cell proliferation, angiogenesis, and cell metastasis and inhibiting cell apoptosis, *NFKB1* is employed as a potential therapeutic target for colon cancer [92]. Furthermore, molecular docking results indicated the stable binding connections between TQ/5-FU and the hub targets, i.e., *MAP2K2*, *CASP3*, *PIK3CA*, *ESR1*, *CYP3A4*, *CYP2C19*, and *CYP2C9*, suggesting that TQ, in combination with 5-FU, may treat colon cancer by modulating these hub targets.

However, alterations in intracellular Ca^2+^ homeostasis are implicated in various stages of the apoptotic signaling cascade [93]. Moreover, it has been reported that Ca^2+^ could act as a messenger for 5-FU to activate p53, where 5-FU may induce apoptosis through a Ca^2+^-calmodulin-dependent pathway, which subsequently modulated DR5-death-inducing signaling complex activity via p53 [94]. Given that calcium carbonate nanoparticles decompose into Ca^2+^ and CO_2_ under acidic conditions, further investigation will be conducted to comprehensively explore the synergistic anti-colon cancer mechanisms of TQ-CaCO_3_ NPs and 5-FU, as the mechanisms may involve the interaction not only between TQ and 5-FU but also between Ca^2+^ and 5-FU. This fact may provide a novel therapeutic intervention point for this combined treatment regimen.

## 5. Conclusion

Taken together, purely aragonite TQ-CaCO_3_ NPs with excellent pH sensitivity, biocompatibility, and sustained-release properties were synthesized by a high-speed homogenizer in this study. The combination of TQ-CaCO_3_ NPs and 5-FU exhibited synergistic anti-colon cancer effects by inhibiting CT26 cell proliferation and migration, inducing cell apoptosis and cell cycle arrest in the G_0_/G_1_ phase, as well as suppressing the CT26 spheroid growth. In other words, TQ-CaCO_3_ NPs can effectively enhance the anti-colon cancer efficacy of 5-FU, indicating that it is possible to reduce the dose of 5-FU while maintaining or improving the effect of 5-FU, thereby decreasing the toxic side effects caused by 5-FU. Furthermore, the underlying therapeutic mechanisms of the combination are possibly associated with multiple molecules and pathways, with a particular emphasis on *ESR1*, *CYP3A4*, *CYP2C19*, *CYP2C9*, *ALOX15*, *MAP2K2*, *CASP3*, *NFKB1*, *PIK3CA*, and *PIK3R1* targets, as well as chemical carcinogenesis-receptor activation and colorectal cancer pathways. These findings may open up new possibilities for the treatment of colon cancer. Therefore, it is imperative to further verify the combination of TQ-CaCO_3_ NPs and 5-FU against colon cancer, including its in vivo therapeutic efficacy and comprehensive therapeutic mechanisms.

## Figures and Tables

**Table 1:** Elemental compositions of CaCO_3_ NPs, TQ-CaCO_3_ NPs, and TQ.

Nanoparticles	C (%)	O (%)	Ca (%)	Na (%)	N (%)	F (%)
CaCO_3_ NPs	16.06	62.42	21.27	0.25	0	0
TQ-CaCO_3_ NPs	17.42	62.00	20.58	0	0	0
TQ	79.58	20.42	0	0	0	0

**Table 2:** Lattice parameters of CaCO_3_ NPs and TQ-CaCO_3_ NPs.

Nanoparticles	a (Å)	b (Å)	c (Å)	α (°)	β (°)	γ (°)	Crystallite Size (Å)	Crystallinity (%)
CaCO_3_ NPs	4.96662	7.96480	5.74860	90	90	90	827	96.35
TQ-CaCO_3_ NPs	4.96283	7.96530	5.74325	90	90	90	848	98.18

**Table 3:** FTIR peak assignments of CaCO_3_ NPs and TQ-CaCO_3_ NPs. FTIR, Fourier transform infrared spectroscopy.

Peak Assignment	Wavenumber (cm^−1^)	
	CaCO_3_ NPs	TQ-CaCO_3_ NPs
In-plane C-O bending	707.47	707.47
Out of plane C-O bending	854.80	851.60
Symmetric C-O stretching	1082.21	1082.21
Asymmetric C-O stretching	1450.53	1447.33
Symmetric C-O stretching; in-plane C-O bending	1783.63	1783.63

**Table 4:** Kinetic analysis of TQ releases from TQ-CaCO_3_ NPs at pH of 7.4, 6.5, and 5.0 using various kinetic models. R^2^, regression coefficient; n, release exponent.

Formulation	pH	Zero-Order Model R^2^	First-Order Model R^2^	Higuchi Model R^2^	Hixon–Crowell Model R^2^	Korsmeyer–Peppas Model	
						R^2^	n
TQ-CaCO_3_ NPs	7.4	0.95013	0.94527	0.98353	0.95486	0.98578	0.57060
	6.5	0.92144	0.95302	0.98363	0.93512	0.98266	0.53670
	5.0	0.90526	0.92793	0.98557	0.92571	0.98765	0.45530

**Table 5:** Molecular docking binding energy of 5-FU and TQ with the hub targets.

Targets	PDB ID	Ligands	Binding Energy (kcal/Mol)
*MAP2K2*	1S9I	5-FU	−4.89
		TQ	−6.12
*CASP3*	3KJF	5-FU	−4.45
		TQ	−4.57
*PIK3CA*	5DXT	5-FU	−4.80
		TQ	−5.40
*ESR1*	6DFN	5-FU	−5.07
		TQ	−6.35
*CYP3A4*	8SO1	5-FU	−5.31
		TQ	−6.79
*CYP2C19*	4GQS	5-FU	−5.08
		TQ	−6.25
*CYP2C9*	7RL2	5-FU	−5.19
		TQ	−6.16

## Data Availability

The data presented in this study are available in the article and Supplementary File.

## Supplementary Materials

The following supporting information can be downloaded at: https://www.mdpi.com/article/10.3390/antiox13091030/s1, Table S1: Single-factor experimental design; Figure S1: Absorption spectrum of TQ with the absorbance peak (252 nm) in ethanol; Figure S2: EDX spectrum of CaCO_3_ NPs; Figure S3: EDX spectrum of TQ-CaCO_3_ NPs; Figure S4: EDX spectrum of TQ.
