# Supplementary material for: 5-Fluorouracil in Combination with Calcium Carbonate Nanoparticles Loaded with Antioxidant Thymoquinone against Colon Cancer: Synergistically Therapeutic Potential and Underlying Molecular Mechanism
Source: Antioxidants (Basel). 2024 Aug 25;13(9):1030. doi: 10.3390/antiox13091030 (PMC11429434; doi:10.3390/antiox13091030)
Supplement: Supplementary file 1 [file antioxidants-13-01030-s001.zip › antioxidants-3132536-supplementary.pdf]

5-Fluorouracil in Combination with Calcium Carbonate Nanoparticles Loaded with Antioxidant Thymoquinone against Colon Cancer: Synergistically Therapeutic Potential and Underlying Molecular Mechanism

Xi Deng [1], Zhongming Yang [1], Kim Wei Chan [1], Norsharina Ismail [1] and Md Zuki Abu Bakar [1, 2,*]

1 Natural Medicines and Products Research Laboratory, Institute of Bioscience, Universiti Putra Malaysia, Serdang 43400, Selangor, Malaysia; dengxi9528@126.com (X.D.); yzm719268164@gmail.com (Z.Y.); chankim@upm.edu.my (K.W.C.); norsharina@upm.edu.my (N.I.)
2 Department of Veterinary Preclinical Science, Faculty of Veterinary Medicine, Universiti Putra Malaysia, Serdang 43400, Selangor, Malaysia
* Correspondence: zuki@upm.edu.my

Table S1. Single-factor experimental design. rpm, revolutions per minute; min, minute.

Factor	Speed (rpm)	Time (min)
Speed	6000	3
	12,000	3
	18,000	3
	24,000	3
Time	24,000	1
	24,000	3
	24,000	5
	24,000	7

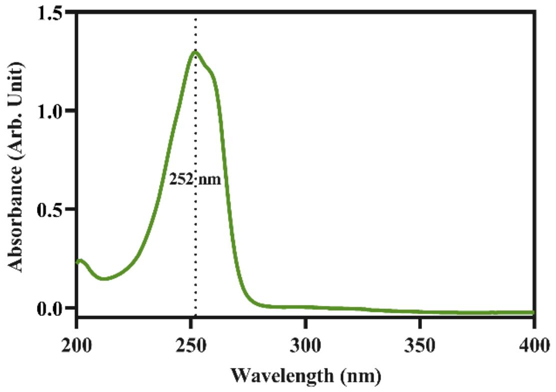

Figure S1. Absorption spectrum of TQ with the absorbance peak (252 nm) in ethanol.

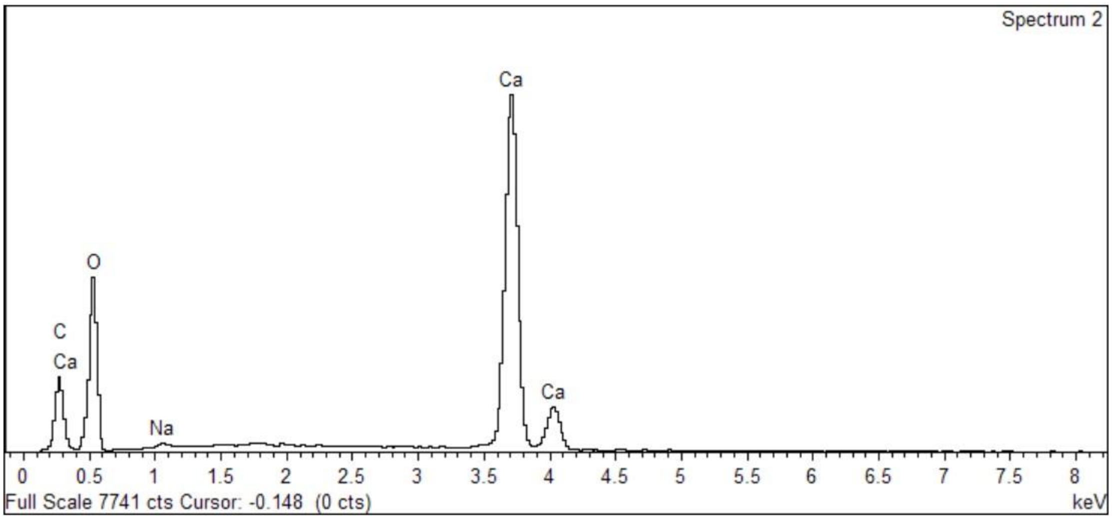

Figure S2. EDX spectrum of $CaCO_3$ NPs. EDX, Energy-dispersive X-ray spectroscopy.

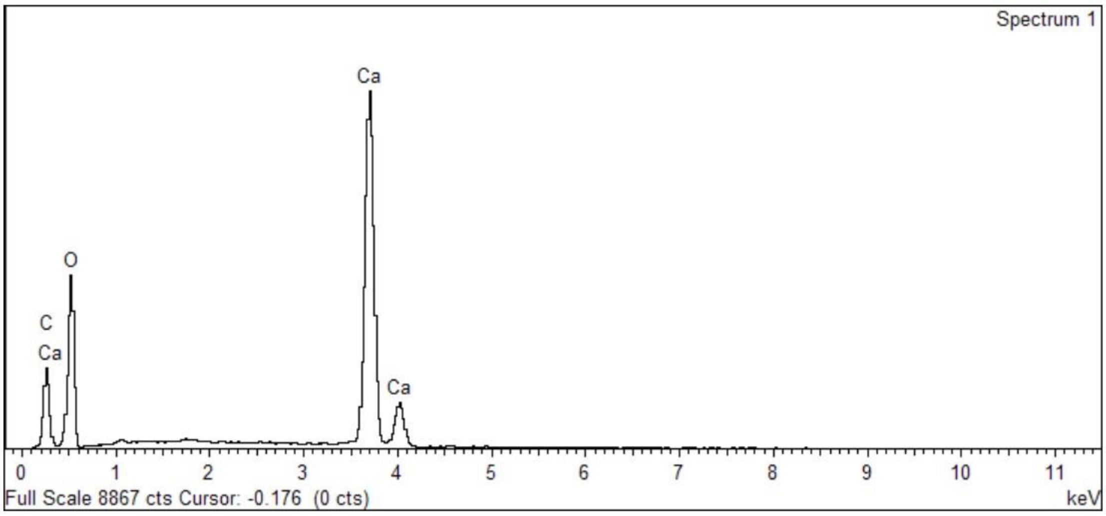

Figure S3. EDX spectrum of TQ-$CaCO_3$ NPs. EDX, Energy-dispersive X-ray spectroscopy.

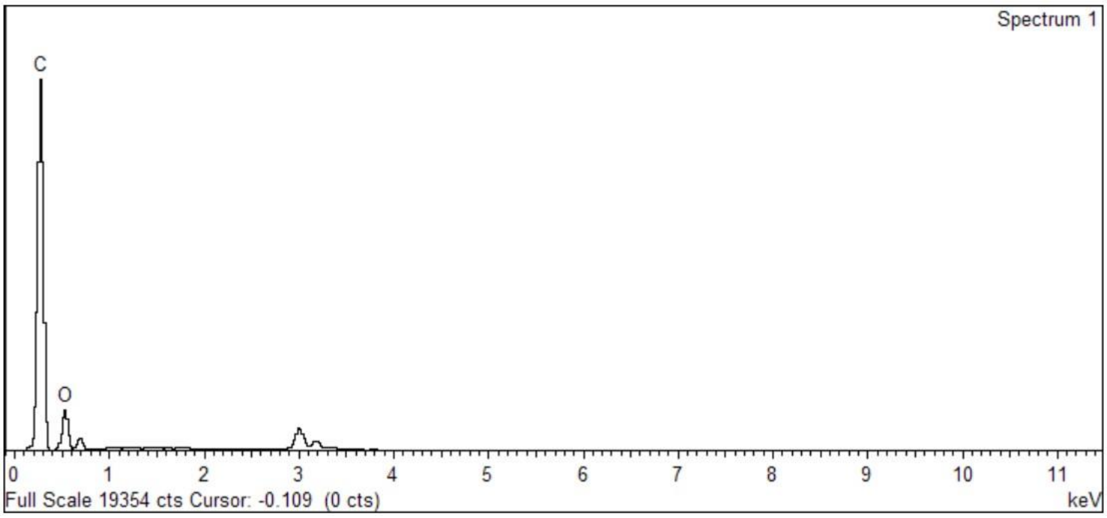

Figure S4. EDX spectrum of TQ. EDX, Energy-dispersive X-ray spectroscopy.
